# Diversity and structure of soil bacterial communities in the Fildes Region (maritime Antarctica) as revealed by 454 pyrosequencing

**DOI:** 10.3389/fmicb.2015.01188

**Published:** 2015-10-28

**Authors:** Neng Fei Wang, Tao Zhang, Fang Zhang, En Tao Wang, Jian Feng He, Hui Ding, Bo Tao Zhang, Jie Liu, Xiang Bin Ran, Jia Ye Zang

**Affiliations:** ^1^Key Lab of Marine Bioactive Substances, The First Institute of Oceanography, State Oceanic AdministrationQingdao, China; ^2^Institute of Medicinal Biotechnology, Chinese Academy of Medical SciencesBeijing, China; ^3^Polar Research Institute of ChinaShanghai, China; ^4^Departamento de Microbiología, Escuela Nacional de Ciencias Biológicas, Instituto Politécnico NacionalMexico, Mexico; ^5^Chemical Engineering Institute, Qingdao UniversityQingdao, China; ^6^Department of Bioengineering and Biotechnology, Qingdao University of Science and TechnologyQingdao, China

**Keywords:** antarctica, 16S rRNA gene, bacterial communities, soil geochemical property, molecular ecology

## Abstract

This study assessed the diversity and composition of bacterial communities in four different soils (human-, penguin-, seal-colony impacted soils and pristine soil) in the Fildes Region (King George Island, Antarctica) using 454 pyrosequencing with bacterial-specific primers targeting the 16S rRNA gene. Proteobacteria, Actinobacteria, Acidobacteria, and Verrucomicrobia were abundant phyla in almost all the soil samples. The four types of soils were significantly different in geochemical properties and bacterial community structure. Thermotogae, Cyanobacteria, Fibrobacteres, Deinococcus-Thermus, and Chlorobi obviously varied in their abundance among the 4 soil types. Considering all the samples together, members of the genera *Gaiella, Chloracidobacterium, Nitrospira, Polaromonas, Gemmatimonas, Sphingomonas*, and *Chthoniobacter* were found to predominate, whereas members of the genera *Chamaesiphon, Herbaspirillum, Hirschia, Nevskia, Nitrosococcus, Rhodococcus, Rhodomicrobium*, and *Xanthomonas* varied obviously in their abundance among the four soil types. Distance-based redundancy analysis revealed that pH (*p* < 0.01), phosphate phosphorus (*p* < 0.01), organic carbon (*p* < 0.05), and organic nitrogen (*p* < 0.05) were the most significant factors that correlated with the community distribution of soil bacteria. To our knowledge, this is the first study to explore the soil bacterial communities in human-, penguin-, and seal- colony impacted soils from ice-free areas in maritime Antarctica using high-throughput pyrosequencing.

## Introduction

Antarctica is isolated geographically from other continents and is one of the most extreme environments on Earth, characterized by low temperatures, large seasonal and diurnal variations in temperature, low precipitation and humidity, low nutrient availability, frequent freeze-thaw and wet-dry cycles, high levels of solar radiation and strong katabatic winds (Wynn-Williams, 1990; Convey, 1996; Cowan, 2009). These extreme conditions support only relatively simple ecosystems, with simple food-web structures comprised of cold adapted microorganisms, plants and animals (Wall and Virginia, 1999). Therefore, Antarctic ecosystems should be particularly sensitive to external disturbances, such as climate warming or human impacts (Bargagli, 2005; Tin et al., 2009).

Terrestrial microbial communities, which are dominant as drivers of soil-borne nutrient cycling, are easily influenced by external disturbances in ice-free areas, as these areas receive impacts directly from animals, such as native marine vertebrates (e.g., penguins and elephant seals). Moreover, in recent decades, climate warming has led to the retreat of glaciers (Cook et al., 2005) and consequently new ice-free areas arise, which further affect populations and distributions of penguins (Ainley et al., 2010) and elephant seals (Hall et al., 2006). Increased human presence in ice-free areas (i.e., research and tourism) imposes additional physical (e.g., foot traffic), chemical (e.g., chemical debris), and biological (e.g., dissemination of non-indigenous species) burdens on local terrestrial ecosystems (Cowan and Tow, 2004; Tin et al., 2009; Cowan et al., 2011). Soil microbial communities may change as increased human and animal activity occurs in newly-formed ice-free areas. Thus, it is crucial to understand the relationships between soil microbial community and impacts of human and animals.

Hitherto, diverse soil bacterial communities have been observed in Antarctic terrestrial ecosystems using traditional isolation methods (Powell et al., 2006; Rinnan et al., 2009; Fan et al., 2013; Zdanowski et al., 2013) and traditional molecular methods (e.g., PCR-DGGE, cloning sequencing, real-time PCR, microarray) (Smith et al., 2006, 2010; Shravage et al., 2007; Yergeau et al., 2007, 2009; Niederberger et al., 2008; Yergeau and Kowalchuk, 2008; Aislabie et al., 2009; Chong et al., 2009, 2010; Soo et al., 2009; Newsham et al., 2010; Ganzert et al., 2011; Ma et al., 2012; Stomeo et al., 2012; Pan et al., 2013; Teixeira et al., 2013). Recently, greater phylogenetic diversity of soil bacterial communities has been found in the Antarctic soils using Roche 454 sequencing (Teixeira et al., 2010; Kim et al., 2012; Lee et al., 2012; Roesch et al., 2012; Tiao et al., 2012; Yergeau et al., 2012), which is able to identify a great number of bacterial sequences and provide an in-depth analysis of soil bacterial diversity. Nevertheless, the vast majority of bacterial community within maritime Antarctic soils has not been characterized adequately. To the best of our knowledge, no study has described bacterial communities in the human-, penguin- and seal-colony soils using high-throughput sequencing.

There is a close correlation between soil bacterial diversity and soil geochemical properties, which is often associated with biological activities including plants and animals. However, the impacts of biological activities are complex and often simultaneously affect soil geochemical properties in the same area. King George Island has ice-free areas of about 8% and weathered soils are derived mainly from volcanic rock (Bölter, 2011). The Fildes Region (62°12′–62°13′S and 58°56′–58°57′W) of the island represents one of the largest ice-free areas in the maritime Antarctica. The human presence and its associated activities may affect the environment around permanent stations in this region. Penguins and elephant seals are also very common in some sites (Braun et al., 2012). Therefore, the Fildes Region is a good place for comparative study of the soil bacterial communities from colonies of human and animals. The aim of this study was to assess the diversity and structure of bacterial communities in four different soils (human-, penguin-, seal-colony soils, and pristine soil) in the Fildes Region using 16S rRNA gene multiplex 454 pyrosequencing. This will allow for a better understanding of soil bacterial community in Antarctic ice-free areas, and contribute to a new perspective on protecting Antarctic pristine ecosystems.

## Materials and methods

### Field site and sampling

**Figure 1:**
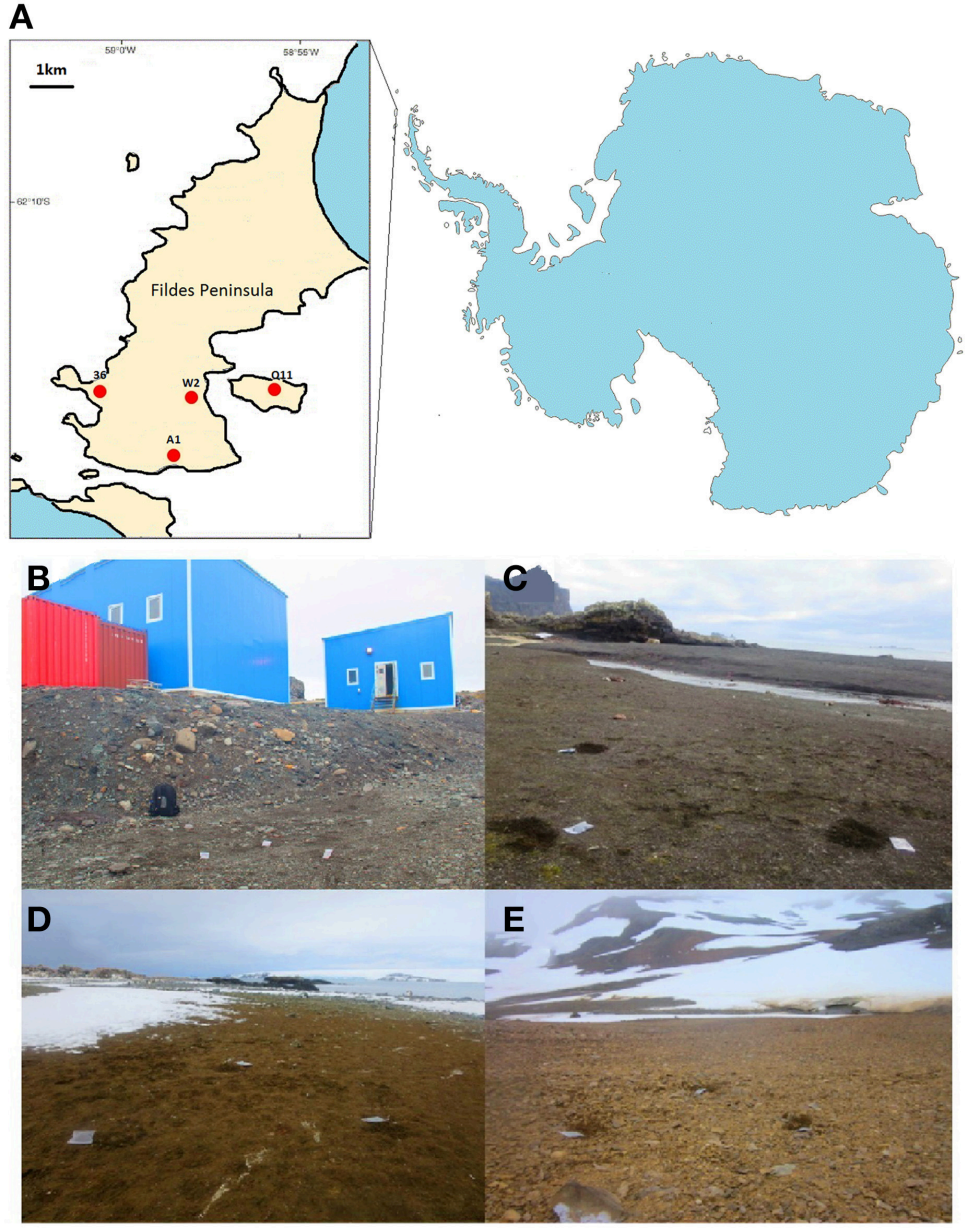
**Locations and images of the four sampling sites in the Fildes Region (sampling sites are marked by red dots)**. **(A)** Map of the four sampling sites; **(B)** Human-colony impacted site (W2); **(C)** Seal-colony impacted site (36); **(D)** Penguin-colony impacted site (Q11); **(E)** Pristine site (A1).

The study area is located in the Fildes Region (62°08′–62°14′S and 59°02′–58°51′W), consisting of the Fildes Peninsula, Ardley Island and adjacent islands, located in the southwestern part of King George Island, South Shetland Islands (Figure 1). Since tertiary lava, pyroclastic rock and volcanic sedimentary rock structured the main body of Fildes Peninsula, volcanic rock erosion and weathering residues has generated very pristine soils there (Zhao and Li, 1994). It is believed that the organic matter (e.g., carbon, nitrogen, phosphates) is transferred into soils by vegetation and animal activities (Bölter, 2011).

Sampling occurred during China's 29th Antarctic expedition in January 2013. Soils (about 50 g) were sampled from soil surface (5 cm) near each other (about 1m apart) in triplicate at four sites (W2, 36, Q11, and A1) (Table 1). Site W2 is typed as human-colony as it is located in the vicinity of Great Wall Cove where many researchers stay for short or long periods at a number of permanent manned research stations. Human presence may impose physical (e.g., foot traffic) and chemical (e.g., chemical dust from burning garbage) effects on the soils of this site. Site 36 is seal-colony as it is located in the vicinity of Horatio Cove where elephant seals (*Mirounga leonina*) densely inhabit. Site Q11 is penguin-colony as it locates in Ardley Island where penguins (*Pygoscelis papua, P. antarctica*, and *P. adeliae*) colonize. Site A1 is pristine as it is located along the south coast of Fildes Peninsula where only scarce plants are found. The landscapes of these sites are shown in Figure 1. These 12 soil samples were placed in sterile plastic bags and taken to the laboratory by air. Samples were then stored at −80°C in the laboratory until further analysis.

**Table 1:** **Locations and geochemical properties of 12 soil samples investigated in the present study**.

**Sample code**	**Soil type**	**Coordinates (N/E)**	**Temperature (°C)**	**Altitude (m)**	**Water content (%)**	**pH**	**Organic C (μg/g)**	**Organic N (μg/g)**	**NH${}_{4}^{+}$-N (μg/g)**	**SiO${}_{4}^{2-}$-Si (μg/g)**	**NO${}_{2}^{-}$-N (μg/g)**	**PO${}_{4}^{3-}$- P (μg/g)**	**NO${}_{3}^{-}$-N (μg/g)**
W2-1	Human-colony impacted soil	62°12.931′–58°57.673′	3.0	3	20.28	7.34	33.38	4.21	2.074	1.169	1.622	3.335	8.799
W2-2					22.96	7.33	33.81	3.75	2.653	1.508	2.243	3.732	13.371
W2-3					18.60	7.44	38.62	4.44	1.989	1.514	2.388	3.776	7.760
Average					20.61A	7.37A	35.27A	4.13A	2.239A	1.397BC	2.084A	3.614A	9.977A
36-1	Seal-colony impacted soil	62°12.682′–59°00.634′	3.9	10	14.98	6.66	1.82	0.24	1.328	3.283	0.352	2.579	0.798
36-2					14.32	6.90	0.69	0.16	1.031	4.401	0.612	1.628	1.322
36-3					13.50	7.20	0.64	0.27	0.833	4.541	0.505	1.664	0.619
Average					14.27B	6.92B	1.05B	0.22B	1.064B	4.075A	0.490C	1.957B	0.913B
Q11-1	Penguin-colony impacted soil	62°12.735′–58°55.102′	2.3	40	15.99	6.33	2.38	0.20	1.309	3.003	0.228	0.966	3.673
Q11-2					17.74	6.68	9.49	0.61	1.349	2.336	0.326	1.530	3.546
Q11-3					17.57	6.69	8.35	0.54	2.169	1.641	0.426	2.007	3.861
Average					17.10B	6.57BC	6.74B	0.45B	1.609AB	2.327B	0.327C	1.504B	3.693B
A1-1	Pristine soil	62°13.764′–58°58.159′	2.0	28	12.58	5.92	0.41	0.01	1.349	1.032	1.042	0.073	1.625
A1-2					16.88	5.77	0.22	0.02	1.161	0.910	1.378	0.065	2.418
A1-3					15.65	5.78	0.66	0.01	0.999	0.909	1.096	0.069	1.608
Average					15.04B	5.82C	0.43B	0.01B	1.170B	0.950C	1.172B	0.069C	1.884B

*Statistical significance was assessed by One-Way ANOVA followed by Tukey's HSD test, and significant differences were accepted when p < 0.05 between the two groups. The letters A and B were used to show statistically significant differences*.

### Soil geochemical analyses

A total of 9 geochemical properties of soil were assessed, including pH, water content, organic carbon, organic nitrogen, ammonium nitrogen (NH${}_{4}^{+}$-N), silicate (SiO${}_{4}^{2-}$-Si), nitrite nitrogen (NO${}_{2}^{-}$-N), phosphate phosphorus (PO${}_{4}^{3-}$-P) and nitrate nitrogen (NO${}_{3}^{-}$-N) (Table 1). Soil pH was measured by adding 10 ml of distilled water to 4 g of soil and recording pH using a pH electrode (PHS-3C, Shanghai REX Instrument Factory, Shanghai, China). Water content was determined as gravimetric weight loss after drying the soil at 105°C until constant weight. Analysis of organic carbon and organic nitrogen was performed using an Elemental Analyzer (EA3000, Euro Vector SpA, Milan, Italy). The other properties were analyzed using a High Performance Microflow Analyzer (QuAAtro, SEAL Analytical GmbH, Norderstedt, Germany).

### DNA extraction

Metagenomic DNA was extracted from an aliquot of 0.25 g of wet soil from each sample using a PowerSoil DNA Isolation Kit (MO BIO Laboratories, San Diego, CA, USA) according to the manufacturer's instructions. The resulting DNA extracts were used for the subsequent PCR and sequencing experiments.

### 454 pyrosequencing

The bacterial hypervariable V3 region of the 16S rRNA genes was amplified using a set of primers designed by adding a 10-nucleotide barcode to primer sets of 533R (5′-TTACCGCGGCTGCTGGCAC-3′) and 8F (5′-AGAGTTTGATCCTGGCTCAG -3′). The 50 μl reaction mixture contained the template DNA (3 μl of sample extract), 8 μl of 5 × buffer, 1 μl of 2.5 nM dNTP, 0.8 μl of Fastpfu (AP221-02, TransGen Biotech Co., Ltd., Beijing, China), 26 μl of ddH_2_O and 0.3 mM of each primer. PCR amplification consisted of an initial denaturation at 95°C for 2 min, 25 cycles of denaturation at 95°C for 30 s, annealing at 56.4°C for 1 min, and extension at 72°C for 30 s, and a final extension at 72°C for 5 min. PCR products were purified using an AxyPrepDNA Gel Extraction Kit (Axygen Biosciences, Corning, NY, USA) according to the manufacturer's instructions. The purified PCR amplicons from each sample were mixed, then pyrosequenced using the 454 GS FLX Titanium Platform (Roche Applied Science, Indianapolis, IN, USA). The raw sequence reads were deposited into the NCBI sequencing read archive under Accession No. SRR1223351.

### Pyrosequencing data treatment

Raw pyrosequencing data were processed using Mothur v. 1.33.3 software (Schloss et al., 2009). Briefly, the sequence libraries were split according to barcode sequence and denoised to avoid diversity overestimation caused by sequencing errors. The resulting sequences met the following criteria: (1) the sequence matches the 533R primer and one of the used barcode sequences; (2) the sequence had no ambiguous bases; (3) the sequence had a length of ≥200 bp; (4) the sequence had an average quality score ≥25; (5) the sequence had homopolymers < 8 bp. These resulting sequences were then simplified by the unique.seqs command and aligned with the SILVA databases v. 115 (Quast et al., 2012). The aligned sequences were clustered by the pre.cluster command (diffs option = 2). Putative chimeric sequences were also detected by the chimera.uchime command and removed from the aligned sequences. The distance matrix between the aligned sequences was generated by the dist.seqs command. In addition, these remaining sequences were clustered to operational taxonomic units (OTUs) at the 3% evolutionary distance by the cluster command (furthest neighbor method). The consensus taxonomy for each OTU was obtained by the classify.otu command with default parameters. Finally, the OTUs that contained only one sequence (singleton OTUs) were removed. These OTUs were used as a basis for calculating alpha-diversity and beta-diversity metrics.

### Statistical analyses

Statistical analysis of OTU richness via rarefaction, Coverage, Chao1 and Shannon's indices were performed using Mothur v. 1.33.3 software (Schloss et al., 2009). One-Way analysis of variance (ANOVA) followed by Tukey's HSD (Honest Significant Difference) test was performed for the soil properties and the diversity parameters to determine the level of significance using Statistical Package for the Social Sciences software (SPSS) v. 17.0. The relationships among the bacterial communities in the 12 soil samples were analyzed by hierarchical clustering analysis using the R v. 3.1.1 statistical software. A Multiple response permutation procedure (MRPP) test was also performed to determine whether the 4 soil types had statistically significantly different bacterial communities using QIIME v. 1.8.0 software (Caporaso et al., 2010). To get better insight into the dissimilarity of soil bacterial communities among the four soil types, a Venn diagram of shared and unique OTUs was performed using Mothur v.1.33.3 software. Furthermore, detailed analyses were performed to visualize the 50 most abundant OTUs and to compare their abundance across the four soil types, including network analysis using Cytoscape v. 2.8 software (Smoot et al., 2011) and heatmap analysis using the Mothur v. 1.33.3 software (Schloss et al., 2009). In order to identify specific taxonomic ranks which are associated with different soil types, the sequence numbers of different taxonomic ranks among the four soil types were analyzed. The relationships between the soil bacterial communities and the geochemical factors were analyzed using distance-based redundancy analysis (db-RDA) and a Monte Carlo permutation test with the R 3.1.1 statistical software.

## Results

### Soil geochemical properties

The highest values of water content, pH, organic C, organic N, NH${}_{4}^{+}$-N, NO${}_{2}^{-}$-N, NO${}_{3}^{-}$-N, and PO${}_{4}^{3-}$-P were recorded at the human-colony impacted site (W2), whereas the lowest values of pH, organic C, organic N, SiO${}_{4}^{2-}$-Si, and PO${}_{4}^{3-}$-P were detected at A1, the pristine site. Soil at penguin colonized ranked behind W2 in pH, water content and concentrations of organic C, organic N, NH${}_{4}^{+}$-N, NO${}_{3}^{-}$-N, and PO${}_{4}^{3-}$-P. The concentration of NO${}_{2}^{-}$-N at site Q11 varied between 0.228 and 0.426 μg/g, which was the lowest among the 4 sites. At elephant seal-occupied site 36, the maximum concentration of SiO${}_{4}^{2-}$-Si (4.075 μg/g) and the minimum concentrations of NH${}_{4}^{+}$-N (1.064 μg/g) and NO${}_{3}^{-}$-N (0.913 μg/g) were recorded (Table 1 and Table S1).

### Pyrosequencing data

A total of 244,765 bacterial sequences and 15,035 OTUs (at the 3% evolutionary distance) were identified in the present study. The sequence number of each samples ranged from 17,574 to 23,120, from which 3503 to 4536 OTUs were recognized at the genetic distances of 3%. The Good's coverage estimator of the OTUs in the samples ranged from 89.38 to 92.85% (Table 2) (rarefaction curves available as Figure S1), indicating that the sequences sufficiently covered the diversity of bacterial populations in the soil samples. Additionally, information on the data being clustered at different thresholds (3, 5, 7, and 10% evolutionary distance) was shown in Table S2. No major differences were shown in the statistical patterns across the samples. For example, at all thresholds, the highest Shannon's index value were observed at penguin-colony impacted soils, followed by pristine soils, human-colony impacted soils, and seal-colony impacted soils.

**Table 2:** **Summary data for pyrosequencing data from the 12 soil samples**.

**Sample code**	**Soil type**	**Number of sequence**	**Number of OTUs^#^**	**Good's coverage estimator (%)**	**Chao 1**	**Shannon's index**
W2-1	Human-colony impacted soil	23,120	4091	92.85	5758	7.19
W2-2		19,515	3631	92.03	5302	7.06
W2-3		20,647	3809	91.80	5827	7.03
Average				92.22A	5629A	7.09AB
36-1	Seal-colony impacted soil	20,382	3825	91.11	6160	7.04
36-2		18,108	3866	89.38	6408	6.86
36-3		21,431	3503	92.30	5524	6.61
Average				90.93A	6030A	6.84B
Q11-1	Penguin-colony impacted soil	22,345	4536	91.25	6750	7.45
Q11-2		19,451	4155	90.61	6199	7.39
Q11-3		17,574	3974	89.90	6062	7.33
Average				90.58A	6337A	7.39A
A1-1	Pristine soil	22,544	4338	92.32	6020	7.38
A1-2		20,093	4310	90.58	6334	7.32
A1-3		19,555	4145	90.48	6240	7.26
Average				91.12A	6198A	7.32A

# *Defined at the cutoff 3% difference in sequence*.

*Statistical significance was assessed by One-Way ANOVA followed by Tukey's HSD test, and significant differences were accepted when p < 0.05 between the two groups. The letters A and B were used to show statistically significant differences*.

### Bacterial diversity and community structure

According to the OTU diversity estimated by Shannon's index, the greatest bacterial diversity was found in the penguin-colony impacted soils (site Q11: 7.33–7.45 with average 7.39), followed by the pristine soils (site A1: 7.26–7.38 with average 7.32), human-colony impacted soils (site W2: 7.03–7.19 with average 7.09) and seal-colony impacted soils (site 36: 6.61–7.04 with average 6.83) (Table 2). The results indicated that there were no significant differences in soil bacterial diversity among the human-colony site W2, penguin-colony site Q11 and pristine site A1. In addition, no significant difference was observed between seal-colony impacted site 36 and human-colony impacted site W2. However, significant differences existed between seal-colony impacted site 36 and the other two sites Q11 and A1 (Table 2).

Twenty phyla and some unidentified bacteria were detected in the present study. Sequences affiliated with Proteobacteria, Actinobacteria, Acidobacteria, and Verrucomicrobia were common in all the four soil types. The most abundant classes in Proteobacteria phylum were Alphaproteobacteria and Betaproteobacteria, followed by Gammaproteobacteria, Deltaproteobacteria, and Epsilonproteobacteria. Analysis of Proteobacteria revealed dominance of the order Rhizobiales (Class Alphaproteobacteria) and Burkholderiales (Class Betaproteobacteria). In Actinobacteria, the most abundant class was Actinobacteridae, followed by Rubrobacteridae, Acidimicrobidae, Coriobacteridae, Nitriliruptoridae, and unclassified Acitinobacteria. The phylum Acidobacteria was represented by bacteria belonging to the classes Sobibacteres, Acidobacteria and unclassified Acidobacteria, whereas Verrucomicrobia was represented by bacteria belonging to the classes Opitutae, Spartobacteria, and Verrucomicrobiae. The predominant genera were *Gaiella* (phylum Actinobacteria), *Chloracidobacterium* (phylum Acidobacteria), *Nitrospira* (phylum Nitrospirae), *Polaromonas* (phylum Proteobacteria), *Gemmatimonas* (phylum Gemmatimonadetes), *Sphingomonas* (phylum Proteobacteria), and *Chthoniobacter* (phylum Verrucomicrobia) (Figure S2 and Table S3).

### The correlation between soil bacterial communities and soil types and environmental factors

**Figure 2:**
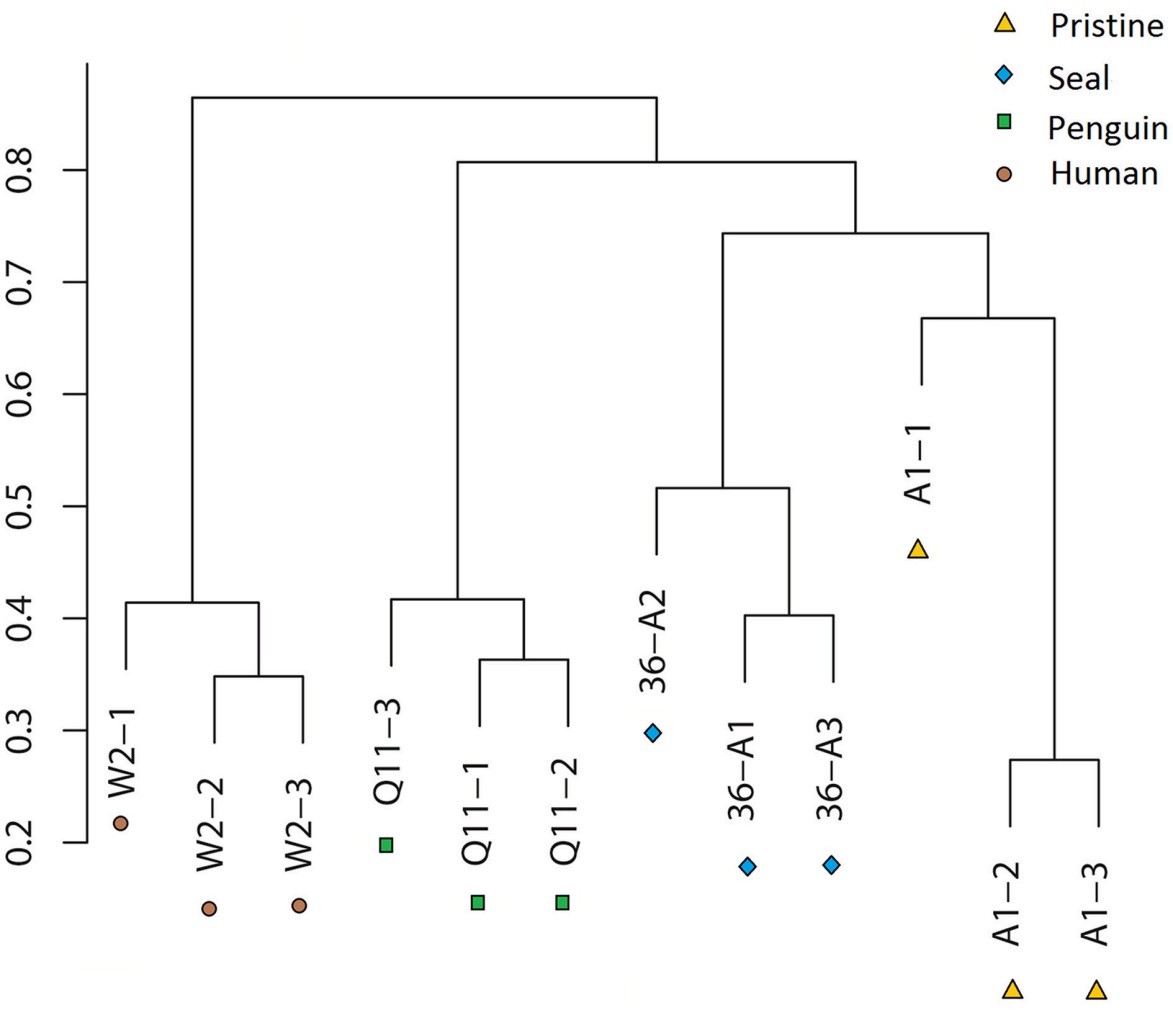
**Clustering analysis of bacterial communities in the 12 soil samples based on OTU abundance-based Bray-Curtis similarity coefficients**.

**Figure 3:**
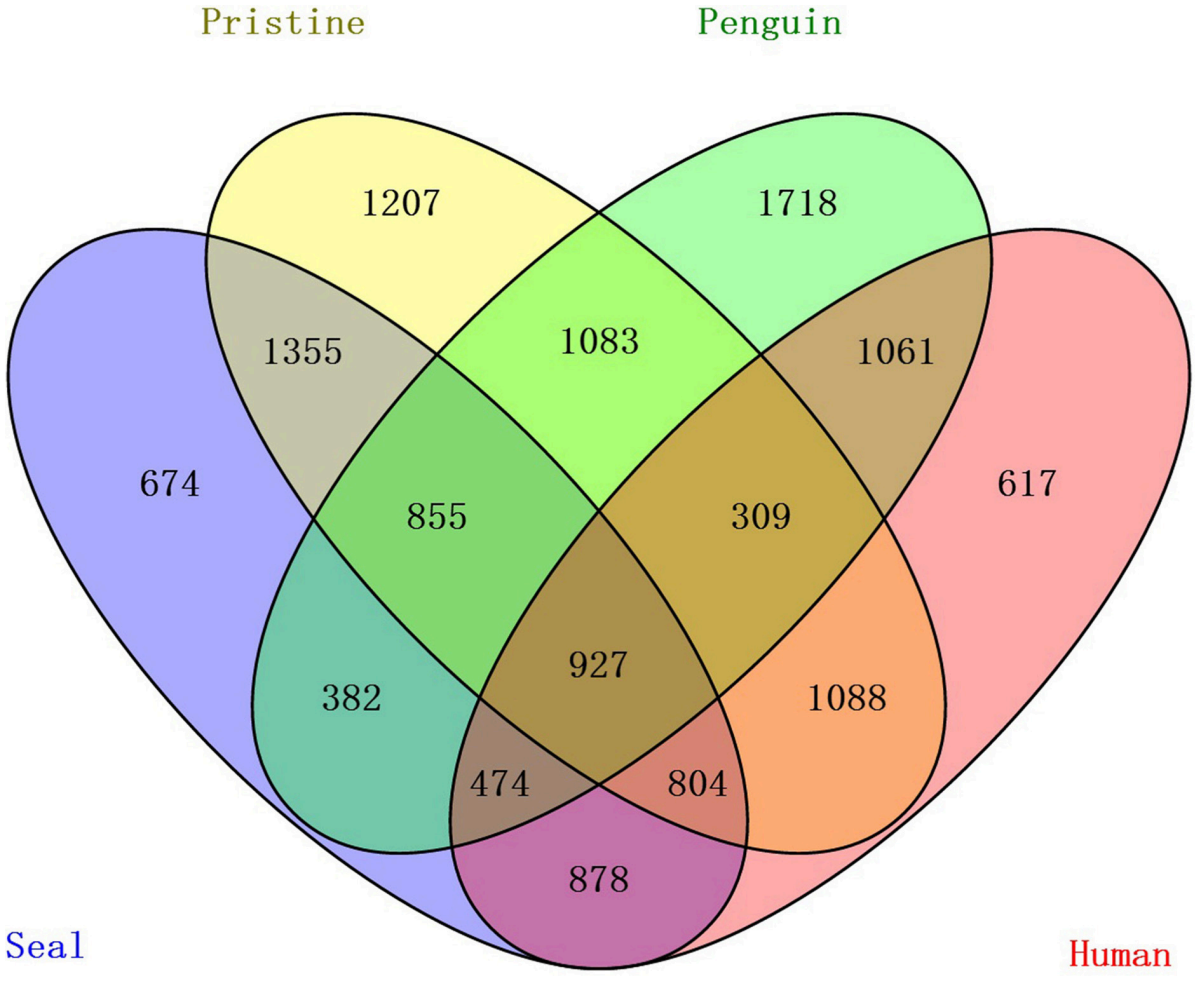
**A Venn diagram displaying the degree of overlap of bacterial OTUs (at the 3% evolutionary distance) among the 4 soil types**.

OTU cluster analysis (Figure 2) revealed that the 12 soil samples were clustered into 4 groups which corresponded to 4 soil types very well. It was shown that the pristine and seal-colony impacted sites were closely related. A MRPP test (*A* = 0.1265, *p* = 0.004) supported that the four soil types (pristine soil, seal-colony impacted soil, penguin-colony impacted soil, and human-colony impacted soil) harbored significantly different bacterial communities (Table S4). A Venn diagram demonstrated that OTUs differed among the four soil types (Figure 3). The number of site-specific OTUs ranged from 3685 (seal-colony soil) to 5589 (human-colony soil). Only 927 in 15,035 OTUs were shared by all four soil types. The number of shared OTUs among soils was low, for example, 3174 between pristine soil and penguin-colony impacted soil; 3941 between pristine soil and seal-colony impacted soil; and 3128 between pristine and human-colony impacted soil.

**Figure 4:**
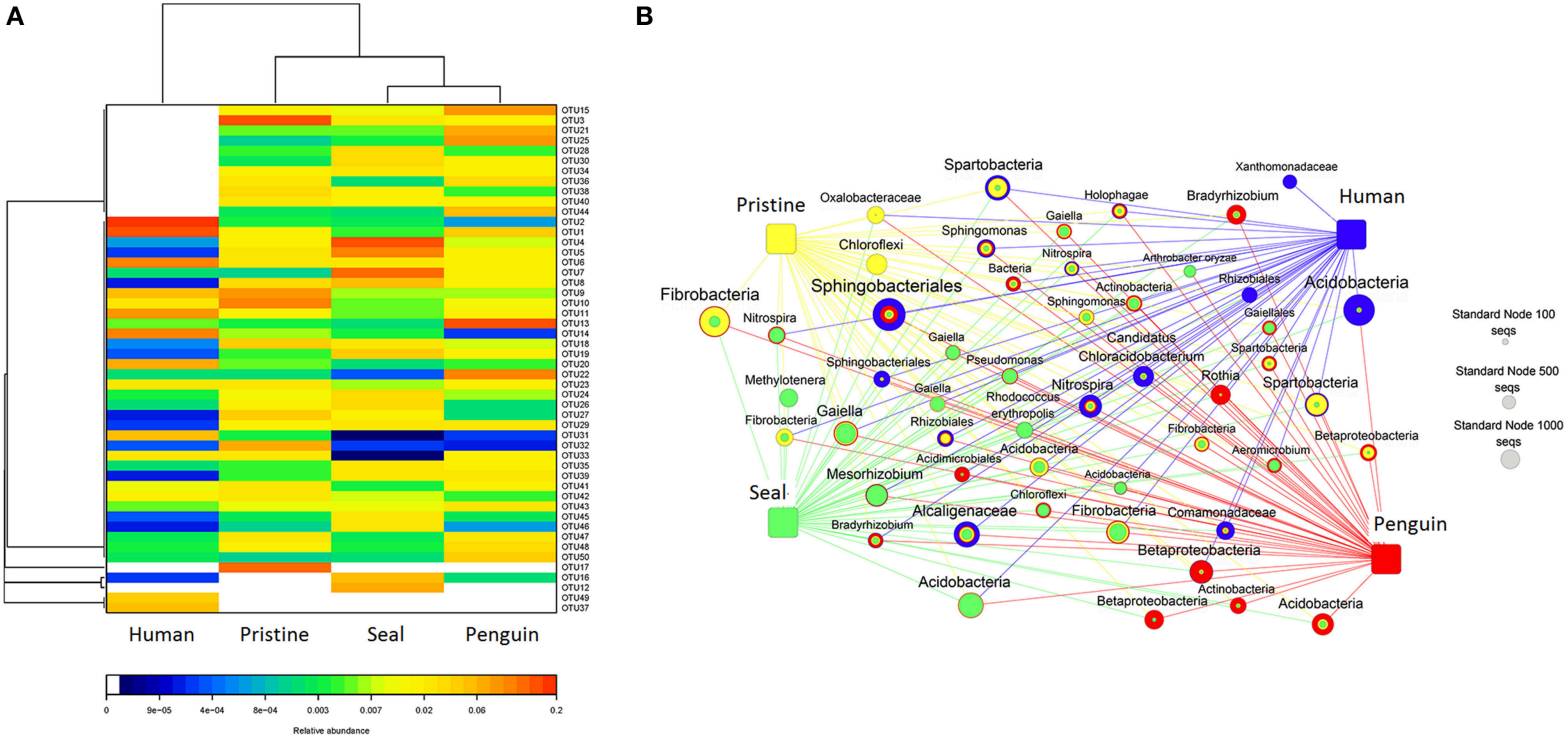
**(A)** A heatmap diagram visualizing the dominant 50 OTUs among the 4 soil types; **(B)** A network diagram showing the dominant 50 OTUs among the 4 soil types.

To get better insight into the differences of soil bacterial community among the four different soil types, we applied network and heatmap analyses of the most abundant 50 OTUs, which highlighted their relative distributions and abundances (Figure 4). As shown in heatmap (Figure 4A) and network diagrams (Figure 4B), the abundance of dominant 50 OTUs differed among the four soil types. The dominant OTUs in each soil type were also different. For example, human-colony impacted soil was dominated by OTU1 (Sphingobacteriales) and OTU2 (Acidobacteria), whereas pristine soil was dominated by OTU3 (Fibrobacteria). Detailed information about sequence number and taxonomy of these 50 OTUs was shown in Table S5.

At different taxonomic ranks, the pristine soil can be distinguished from other animal-colony impacted soils, as shown in Table 3. For example, at the phylum rank, members of Thermotogae was detected than in the pristine soil, but not observed in the seal-, penguin-, human-colony impacted soils. At the class rank, the sequence number of Ignavibacteria in the seal-, penguin-, human-colony impacted soils was much higher (above a five-fold change) than that in the pristine soil. Similarly, the bacterial community composition in the four soil types can be distinguished at ranks of order, family, genus, and species. For examples, members of the genera *Chamaesiphon, Herbaspirillum, Hirschia, Nevskia, Nitrosococcus, Rhodococcus, Rhodomicrobium*, and *Xanthomonas* varied obviously in their abundance between pristine soil and animal-colony impacted soils.

**Table 3:** **The taxonomic ranks in which obvious fold differences occur between pristine soil and human-, seal-, penguin-colony impacted soils**.

**Taxonomic rank**	**Name**	**Fold difference between soil types**					
		**Pristine/Human**	**Human/Pristine**	**Pristine/Seal**	**Seal/Pristine**	**Pristine/Penguin**	**Penguin/Pristine**
Phylum	Fibrobacteres	13.42^*^	0.07	1.17	0.86	2.87	0.07
Phylum	Cyanobacteria	11.14^*^	0.09	7.39^*^	0.14	2.68	0.09
Phylum	Thermotogae	>8.23^*^	–	>8.23^*^	–	>8.23^*^	–
Phylum	Deinococcus-Thermus	0.47	2.11	10.01^*^	0.1	1.82	2.11
Phylum	Chlorobi	0.16	6.25^*^	1.15	0.87	0.91	6.25^*^
Class	Fibrobacteria	13.42^*^	0.07	1.17	0.86	2.87	0.07
Class	Deinococci	0.47	2.11	10.01^*^	0.1	1.82	2.11
Class	Chlorobia	0.16	6.23^*^	1.21	0.82	0.91	6.23^*^
Class	Ignavibacteria	0.11	8.86^*^	0.19	5.25^*^	2.85	8.86^*^
Order	Chromatiales	198.01^*^	0.01	3.15	0.32	10.91	0.01
Family	Chromatiaceae	175.51^*^	0.01	3.16	0.32	10.74	0.01
Family	Hyphomonadaceae	–	>135.03^*^	–	–	–	>135.03^*^
Genus	*Chamaesiphon*	>234.01^*^	–	34.86	0.03	243.52^*^	–
Genus	*Nitrosococcus*	>175.51^*^	–	3.16	0.32	10.74	–
Genus	*Herbaspirillum*	148.26^*^	0.01	2.53	0.4	0.98	0.01
Genus	*Rhodococcus*	–	>1.8	–	>832.44^*^	–	>1.8
Genus	*Xanthomonas*	0.01	114.73^*^	0.13	7.87	0.14	114.73^*^
Genus	*Hirschia*	–	>122.43^*^	–	–	–	>122.43^*^
Genus	*Rhodomicrobium*	0.01	133.93^*^	0.64	1.57	0.01	133.93^*^
Genus	*Nevskia*	–	>144.03^*^	–	–	–	>144.03^*^
Species	*Arthrobacter oryzae*	45.7	0.02	>123.41^*^	–	16.05	0.25

The sequence number of each soil type was normalized to that in per 10,000 sequences. –, calculation is not available as no sequence was obtained; >, underestimated as 1 was assigned if no sequence was obtained;

* *indicating obvious fold difference >5 (phylum and class) and >100 (order, family, genus, and species)*.

**Figure 5:**
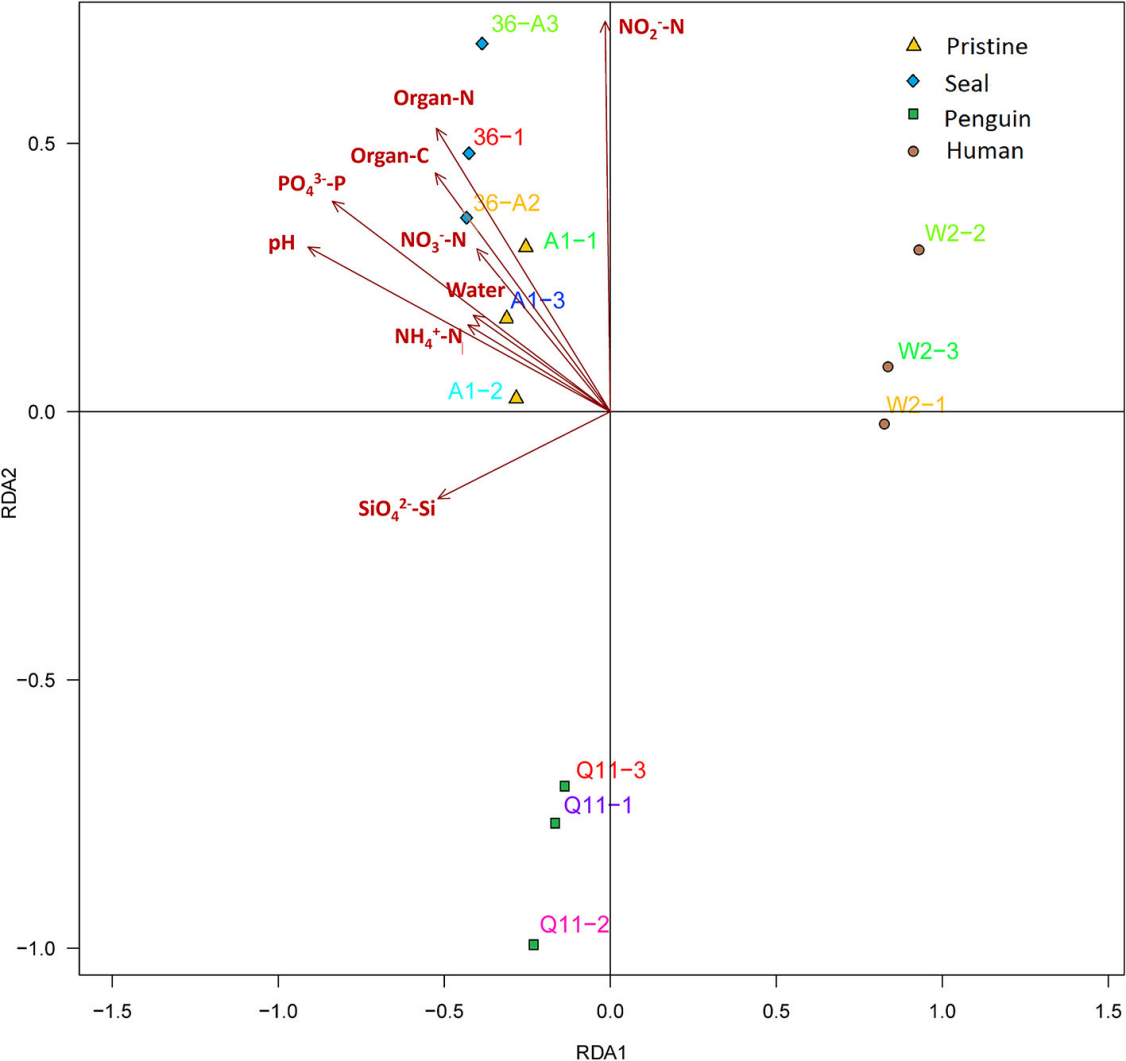
**Distance-based redundancy analysis to show correlations between the bacterial communities and geochemical properties of the 4 soil types**. The arrows represent geochemical factors measured. The 12 soil samples are labeled with unique sampling codes.

Distance-based redundancy analysis (db-RDA) (Figure 5) and Monte Carlo permutation test (Table 4) were performed to examine the relationship between the nine soil geochemical factors and bacterial community composition. The combination of the nine environmental factors showed a significant correlation with soil bacterial community structure (*F* = 6.163631, *p* = 0.001). These factors explained 96.52% of the soil community variation, while 3.48% of the variation was not explained by any of the selected nine environmental parameters. Among the selected geochemical factors, pH (*r*^2^ = 0.9220, *p* < 0.01), PO${}_{4}^{3-}$-P (*r*^2^ = 0.8527, *p* < 0.01), organic C (*r*^2^ = 0.4712, *p* < 0.05), and organic N (*r*^2^ = 0.5456, *p* < 0.05) were important geochemical factors that correlated with the soil bacterial community composition in this region. However, the other five environmental factors, including water content, NH${}_{4}^{+}$-N, SiO${}_{4}^{2-}$-Si, NO${}_{2}^{-}$-N, and NO${}_{3}^{-}$-N, were not significantly correlated with soil bacteria community composition (Table 4).

**Table 4:** **A Monte Carlo permutation test of environmental factors and soil bacterial community composition**.

	**RDA1**	**RDA2**	***r*^2^**	***P*-value**
Water content	−0.9026635	0.4303471	0.2024	0.364
Organic carbon	−0.7499563	0.6614874	0.4712	0.037^*^
Organic nitrogen	−0.6901439	0.7236722	0.5456	0.013^*^
pH	−0.9361896	0.3514954	0.9220	0.001^**^
NH${}_{4}^{+}$-N	−0.9214419	0.3885161	0.2101	0.378
SiO${}_{4}^{2-}$-Si	−0.9694442	−0.2453119	0.2899	0.213
NO${}_{2}^{-}$-N	0.0052103	0.9999864	0.5022	0.053
PO${}_{4}^{3-}$-P	−0.8913095	0.4533954	0.8527	0.001^**^
NO${}_{3}^{-}$-N	−0.7868960	0.6170857	0.2857	0.219

* *Correlation is significant at the 0.05 level*.

** *Correlation is significant at the 0.001 level*.

*P-values based on 999 permutations*.

## Discussion

Despite geographic isolation and extreme environmental conditions, diverse soil bacterial communities were observed in this study. The high level of Shannon diversity indices (*H*′ = 6.61–7.45) and the identification of 3503–4536 OTUs suggest the presence of a surprisingly high diversity of soil bacterial communities in the Fildes Region. By comparison, using 454 pyrosequencing, Teixeira et al. (2010) reported a Shannon diversity of 4.87–5.71 and 552–732 OTUs for bacterial communities in rhizosphere soil from Antarctic vascular plants in King George Island. The observed OTU number was much higher than that in previous studies, which may due to the higher sequence numbers of each sample in this study (17,574–23,120 reads). Teixeira et al. (2010) only sequenced 1821–2918 reads per sample for soil bacterial communities in Antarctica.

The relative abundances of the dominant phyla Proteobacteria, Actinobacteria, and Acidobacteria, as well as the identification of Verrucomicrobia as one of the dominant phyla in the soils of the Fildes Region in this study, was somewhat different from those previously described for soils in the King George Island using pyrosequencing (Teixeira et al., 2010; Kim et al., 2012; Roesch et al., 2012). Based on the 16S rDNA pyrosequencing data, Firmicutes, Proteobacteria, Bacteroidetes, Acidobacteria and Actinobacteria were the dominant soil bacterial phyla in Antarctica, including rhizosphere soil from vascular plants (Teixeira et al., 2010), ornithogenic and mineral soils (Kim et al., 2012), soils in exposed control site and in seal-covered site (Tiao et al., 2012), and soils from other different sites (Lee et al., 2012; Roesch et al., 2012; Yergeau et al., 2012). These differences may be due to the biotic and abiotic factors in the Antarctic soils. Some biotic factors were previously found to be related to the soil bacterial community, such as presence of plants (Teixeira et al., 2010, 2013), birds (Teixeira et al., 2013), penguins and seals (Ma et al., 2012), and mummified seals (Tiao et al., 2012).

In this study, significant differences in geochemical properties and bacterial communities were observed among the four soil types, including pristine soil, seal-, penguin-, human-colony impacted soils. Interestingly, the seal-colony impacted soil (36) was most similar to the pristine soil (A1), which suggests that seals may impose less impact on soil bacterial community than penguins or humans. The population quantity of seals on the colony site (36) is obviously lower than that of penguins on the colony site (Q11), and seals spent relatively little time living on land as compared with humans. By contrast, large populations of penguins colonized on the site (Q11), whereas humans have imposed physical and chemical impacts on the site (W2) for a long time.

Although soil bacterial diversity and richness indices did not vary much among the four different sites, some specific groups varied a lot. Members of the genera *Chamaesiphon, Herbaspirillum, Hirschia, Nevskia, Nitrosococcus, Rhodococcus, Rhodomicrobium*, and *Xanthomonas* varied obviously in their abundance between pristine soil and animal-colony impacted soils. Some of these genera were specifically associated with a given site and further explanations are needed. For example, the genus *Nevskia*, which was highly related to ammonium (Kangatharalingam and Priscu, 1993), was only detected in human- and penguin-colony impacted soils. One cause may be that content of NH${}_{4}^{+}$-N was relatively high in human- and penguin-colony impacted soils as compared with pristine and seal-colony impacted soils.

The combined nine geochemical factors showed a significant correlation with soil bacterial community structure in this region. In this study, pH was the best predictor of soil bacterial community composition. Furthermore, content of PO${}_{4}^{3-}$-P, organic C, and organic N showed significant and positive correlation with the soil bacterial community composition. It is estimated that pH and nutrient content directly alter bacterial community composition by imposing a physiological constraint on survival and growth of soil bacteria. In the previous studies, Ganzert et al. (2011) reported that the soil bacterial community composition was most affected by total carbon and total nitrogen contents and soil physical factors such as moisture, but not pH, whereas Stomeo et al. (2012) revealed K, C, Ca, and moisture influenced the distribution and structure of microbial populations in Antarctic Valley. In contrast, Newsham et al. (2010) found that different levels of C, N, and P have only a minor effect on the bacterial community composition of maritime Antarctic soils. In this study, the other five geochemical factors, including water content, NH${}_{4}^{+}$-N, SiO${}_{4}^{2-}$-Si, NO${}_{2}^{-}$-N, and NO${}_{3}^{-}$-N, showed positive but no significant correlation with soil bacterial community composition. Particularly, the high level of SiO${}_{4}^{2-}$-Si in seal and penguin occupied soils should be related to the food chain of these two marine animals and may have an indirect effect on soil bacterial communities in these two soil types. Silicon is an essential nutrient for marine organisms, such as diatoms, radiolaria and sponges, and its cycling is coupled with CO_2_ fixation (Sarmiento and Gruber, 2006).

The selected nine geochemical factors explained the majority of the variation of the soil bacterial community; however, there was the minor variation that remained unexplained in this study, suggesting that there were many unmeasured factors. It is estimated that penguins and elephant seals both possess rich gut bacterial community (Nelson et al., 2013), which might influence the soil bacterial communities by guano and excreta. Some bacterial species might be introduced into Antarctic soils by human activities, as many non-indigenous plant and animal species did in Antarctica (Tin et al., 2009).

Our results did not prove the causal relationship between impacts of seal, penguin, human and soil geochemical factors. The question is as follows: can human and animal activities directly cause the observed geochemical differences in the Antarctic soils described in this paper? Some previous studies reported that human and animal activities could affect geochemical property and microbial community in the Antarctic soils. Human impacts were found to affect soil geochemical properties and soil bacterial diversity from sites around Casey Station in East Antarctica (Chong et al., 2010). Intense penguin's activity in ice-free areas led to highly ornithogenic soils (high N and P concentrations and acidic pH) (Simas et al., 2007). Organic matter could be added to the soil in the forms of penguin guano, feathers, eggshells and bird remains (Aislabie et al., 2009), whereas elephant seal excreta was also an important source of nutrients to Antarctic soils (Ma et al., 2012, 2013).

Antarctic soil microbial communities are associated with the environmental conditions and thereby can serve as a sensitive environmental indicator. In the future, climate warming in Antarctica will result in more ice-free areas, expansion of flora and fauna, change of soil environmental conditions, which will likely influence soil microbial communities. Climate change on King George Island has already resulted in substantial and rapid changes in the environment in the years 1948–2011, which posing a great threat to the local ecosystem (Kejna et al., 2013). The relationship between soil microbial communities and climate warming should be clarified in further studies. The other remaining question is as follows: what are the possible functional roles of these soil microbial communities in the Antarctic ice-free area? Perhaps the use of other molecular tools (e.g., shotgun metagenomic sequencing or Geochip) will allow us to clarify and establish connections between microbial structure and its ecosystem functioning.

In addition, one contribution of this study is an increase in the knowledge about the bacterial diversity in four different types of Antarctic soil (human-, penguin-, seal-colony impacted soils, and pristine soil). Based on these new data about the great diversity of bacteria in these different Antarctic soils, it should be able to isolate particular bacterial species from these Antarctic soils, and reveal their interactions with many organisms in the future.

### Conflict of interest statement

The authors declare that the research was conducted in the absence of any commercial or financial relationships that could be construed as a potential conflict of interest.
