# Supplementary material for: Diversity and structure of soil bacterial communities in the Fildes Region (maritime Antarctica) as revealed by 454 pyrosequencing
Source: Front Microbiol. 2015 Oct 28;6:1188. doi: 10.3389/fmicb.2015.01188 (PMC4623505; doi:10.3389/fmicb.2015.01188)
Supplement: Supplementary file 2 [file Table2.PDF]

Table S2 | Information on the pyrosequencing data being clustered at different thresholds (3, 5, 7, and 10% evolutionary distance).

	OTUs (97%)	Chao 1	Shannon	OTUs (95%)	Chao 1	Shannon	OTUs (93%)	Chao 1	Shannon	OTUs (90%)	Chao 1	Shannon
W2-1	4091	5758	7.19	3481	4857	6.96	2893	3969	6.69	2235	2952	6.40
W2-2	3631	5302	7.06	3089	4383	6.82	2576	3574	6.56	2027	2638	6.28
W2-3	3809	5827	7.03	3163	4558	6.80	2658	3770	6.55	2059	2814	6.27
Average		5629A	7.09AB		4599A	6.86A		3771A	6.60A		2801A	6.32A
36-1	3825	6160	7.04	3138	4728	6.69	2582	3703	6.41	1969	2717	6.04
36-2	3866	6408	6.86	3156	4856	6.52	2636	3888	6.29	1995	2829	5.92
36-3	3503	5524	6.61	2849	4323	6.29	2327	3364	6.04	1818	2541	5.71
Average		6030A	6.84B		4635A	6.50B		3651A	6.25B		2695A	5.89B
Q11-1	4536	6750	7.45	3680	5177	7.13	2986	4009	6.85	2150	2810	6.42
Q11-2	4155	6199	7.39	3368	4947	7.08	2728	3849	6.81	1966	2656	6.39
Q11-3	3974	6062	7.33	3246	4726	7.03	2673	3729	6.77	1951	2637	6.38
Average		6337A	7.39A		4950A	7.08A		3862A	6.81A		2701A	6.40A
A1-1	4338	6020	7.38	3546	4669	7.07	2961	3767	6.79	2244	2862	6.40
A1-2	4310	6334	7.32	3545	4944	7.04	2967	4008	6.79	2236	2919	6.42
A1-3	4145	6240	7.26	3409	4937	6.96	2860	4007	6.71	2175	2870	6.35
Average		6198A	7.32A		4850A	7.02A		3927A	6.76A		2883A	6.39A

Statistical significance was assessed by one-way ANOVA followed by Tukey 's HSD test, and significant differences were accepted when $p<0.05$ between the two groups. The letters A and B were used to show statistically significant differences.
