# Supplementary material for: Diversity and structure of soil bacterial communities in the Fildes Region (maritime Antarctica) as revealed by 454 pyrosequencing
Source: Front Microbiol. 2015 Oct 28;6:1188. doi: 10.3389/fmicb.2015.01188 (PMC4623505; doi:10.3389/fmicb.2015.01188)
Supplement: Supplementary file 3 [file Table3.PDF]

Table S3 | Information on the sequence number of different taxonomic ranks among the 12 soil samples.

	Taxonomic rank	Name	36-1	36-2	36-3	A1-1	A1-2	A1-3	Q11-1	Q11-2	Q11-3	W2-1	W2-2	W2-3	Total
1	superkingdom	Bacteria	20382	18108	21431	22544	20093	19555	22345	19451	17574	23120	19515	20647	244765
2	kingdom	UnClassified	20382	18108	21431	22544	20093	19555	22345	19451	17574	23120	19515	20647	244765
3	phylum	Bacteroidetes	817	758	610	1526	1109	1213	1450	1189	1230	2837	1634	1966	16339
3	phylum	Acidobacteria	2526	3692	2265	3119	2528	2522	3737	2884	2908	3500	3554	3582	36817
3	phylum	Fibrobacteres	1191	306	1235	1011	1225	1108	465	331	305	105	82	65	7429
3	phylum	Actinobacteria	5962	4238	6264	3620	2027	1841	3893	3130	3089	2253	2059	2197	40573
3	phylum	Proteobacteria	4039	4712	6460	5590	5789	5639	6056	6550	4623	6081	4943	5389	65871
3	phylum	Verrucomicrobia	753	929	666	2446	2233	2365	2178	1848	1781	2362	1595	2105	21261
3	phylum	Nitrospirae	590	379	384	502	304	316	406	290	276	434	559	507	4947
3	phylum	Chloroflexi	2300	1158	1321	1026	1372	1273	1481	905	711	1414	1674	1283	15918
3	phylum	UnClassified	658	699	1036	833	1183	1115	792	868	680	940	939	1131	10874
3	phylum	Chlorobi	81	88	70	96	97	90	87	87	118	807	523	499	2643
3	phylum	Armatimonadetes	131	137	80	190	309	273	165	132	180	354	228	218	2397
3	phylum	Cyanobacteria	67	130	57	1175	435	421	145	124	456	100	38	31	3179
3	phylum	Gemmatimonadetes	570	365	412	697	728	720	647	524	541	909	899	893	7905
3	phylum	Planctomycetes	571	462	445	638	666	600	740	510	508	847	670	666	7323
3	phylum	Firmicutes	90	24	113	22	30	24	44	41	116	115	52	59	730
3	phylum	Fusobacteria	4	6	2	10	5	4	13	11	21	8	3	2	89
3	phylum	Deinococcus-Thermus	0	2	0	9	6	2	2	3	3	14	6	15	62
3	phylum	Elusimicrobia	28	23	11	25	47	29	44	24	28	39	54	38	390
3	phylum	Thermotogae	0	0	0	9	0	0	0	0	0	0	0	0	9
3	phylum	Lentisphaerae	3	0	0	0	0	0	0	0	0	1	2	1	7
3	phylum	Spirochaetes	1	0	0	0	0	0	0	0	0	0	1	0	2

4	subphylum	UnClassified	20382	18108	21431	22544	20093	19555	22345	19451	17574	23120	19515	20647	244765
5	class	Sphingobacteriia	732	682	539	1371	1004	1105	1358	1086	1137	2594	1539	1802	14949
5	class	Acidobacteria	1926	3289	1796	2521	2016	2026	3005	2285	2347	3253	3074	3293	30831
5	class	Fibrobacteria	1191	306	1235	1011	1225	1108	465	331	305	105	82	65	7429
5	class	UnClassified	8413	4870	6769	5953	4776	4419	5639	4387	4095	3851	3703	3808	60683
5	class	Betaproteobacteria	2259	2723	3364	2384	3242	3323	2285	3254	1936	2328	1845	2125	31068
5	class	Alphaproteobacteria	1143	1264	2336	1802	1462	1306	2597	2247	1841	1823	1818	1778	21417
5	class	Spartobacteria	514	689	460	2140	1866	2046	1774	1517	1472	1859	1246	1653	17236
5	class	Nitrospira	590	379	384	502	304	316	406	290	276	434	559	507	4947
5	class	Actinobacteria	759	1438	2038	1051	874	776	937	934	1225	1051	689	952	12724
5	class	Gammaproteobacteria	379	496	542	755	409	480	388	405	352	1079	543	730	6558
5	class	Holophagae	566	377	455	575	507	489	711	585	548	246	469	283	5811
5	class	Deltaproteobacteria	241	207	202	612	608	477	735	589	444	797	690	722	6324
5	class	Chlorobia	72	88	64	96	96	89	87	87	117	802	502	498	2598
5	class	Gemmatimonadetes	570	365	412	697	728	720	647	524	541	909	899	893	7905
5	class	Chloroflexi	24	50	30	75	94	80	71	43	35	202	368	154	1226
5	class	Anaerolineae	145	196	92	110	77	79	114	79	45	308	377	282	1904
5	class	Flavobacteria	39	35	36	51	29	30	45	33	31	58	37	129	553
5	class	Planctomycetacia	370	262	317	286	264	237	296	170	181	411	303	281	3378
5	class	Clostridia	23	14	42	13	13	8	29	31	65	101	50	50	439
5	class	Bacilli	67	10	71	9	17	16	15	10	51	14	2	9	291
5	class	Ktedonobacteria	18	27	21	31	45	44	112	81	21	126	105	92	723
5	class	Thermomicrobia	59	47	39	57	13	15	58	25	19	87	120	81	620
5	class	Phycisphaerae	142	151	93	197	207	177	374	284	290	283	261	248	2707
5	class	Verrucomicrobiae	31	34	18	36	26	24	23	13	35	47	22	28	337

5	class	Fusobacteria	4	6	2	10	5	4	13	11	21	8	3	2	89
5	class	Opitutae	11	16	17	29	26	22	55	44	43	72	41	56	432
5	class	Deinococci	0	2	0	9	6	2	2	3	3	14	6	15	62
5	class	Cytophagia	27	32	21	75	60	67	22	48	37	74	41	17	521
5	class	Bacteroidia	16	8	12	17	13	8	25	21	21	94	16	16	267
5	class	Ignavibacteria	9	0	6	0	1	1	0	0	1	5	21	1	45
5	class	Caldilineae	13	22	7	35	32	30	11	8	9	45	29	37	278
5	class	Elusimicrobia	28	23	11	25	47	29	44	24	28	39	54	38	390
5	class	Thermotogae	0	0	0	9	0	0	0	0	0	0	0	0	9
5	class	Epsilonproteobacteria	0	0	0	0	1	2	2	2	2	1	0	2	12
5	class	Spirochaetes	1	0	0	0	0	0	0	0	0	0	1	0	2
6	subclass	UnClassified	20382	18108	21431	22544	20093	19555	22345	19451	17574	23120	19515	20647	244765
7	order	Sphingobacteriales	732	682	539	1371	1004	1105	1358	1086	1137	2594	1539	1802	14949
7	order	UnClassified	13159	11049	12672	13541	11786	11329	13397	11081	10904	10981	9903	10554	140356
7	order	Burkholderiales	754	1199	1850	1193	2043	2238	399	1324	398	1284	908	1211	14801
7	order	Rhizobiales	576	642	1802	716	598	500	1566	1422	1123	1011	1190	1025	12171
7	order	Nitrospirales	590	379	384	502	304	316	406	290	276	434	559	507	4947
7	order	Methylophilales	380	758	432	47	7	11	49	164	70	3	1	4	1926
7	order	Sphingomonadales	404	430	408	823	593	511	499	397	325	334	234	316	5274
7	order	Pseudomonadales	188	233	336	63	67	118	13	139	13	53	24	50	1297
7	order	Xanthomonadales	138	209	150	478	272	263	361	226	308	963	466	633	4467
7	order	Acidimicrobiales	508	371	358	409	310	256	920	606	571	551	457	491	5808
7	order	Nitrosomonadales	532	237	545	427	479	443	346	342	200	271	255	236	4313
7	order	Rhodospirillales	132	159	101	197	209	222	427	358	293	363	280	284	3025
7	order	Myxococcales	141	134	140	520	456	380	616	476	352	570	449	469	4703

7	order	Chlorobiales	72	88	64	96	96	89	87	87	117	802	502	498	2598
7	order	Gemmatimonadales	556	359	405	677	717	706	633	511	535	885	866	873	7723
7	order	Chloroflexales	22	41	24	53	73	65	69	41	35	196	364	151	1134
7	order	Anaerolineales	145	196	92	110	77	79	114	79	45	308	377	282	1904
7	order	Solirubrobacterales	565	352	444	384	223	208	215	164	116	341	309	366	3687
7	order	Chromatiales	23	9	24	160	13	21	0	14	2	0	0	0	266
7	order	Flavobacteriales	39	35	36	51	29	30	45	33	31	58	37	129	553
7	order	Caulobacterales	13	17	15	8	32	30	94	49	78	64	69	88	557
7	order	Planctomycetales	370	262	317	286	264	237	296	170	181	411	303	281	3378
7	order	Clostridiales	23	14	42	13	13	8	29	31	65	101	50	50	439
7	order	Bacillales	57	9	64	3	7	8	2	2	8	0	2	4	166
7	order	Hydrogenophilales	0	2	7	3	4	2	9	2	8	23	33	28	121
7	order	Phycisphaerales	40	37	20	27	25	18	47	36	33	55	82	73	493
7	order	Syntrophobacterales	0	0	0	0	3	0	0	0	0	39	9	16	67
7	order	Verrucomicrobiales	31	34	18	36	26	24	23	13	35	47	22	28	337
7	order	Acidobacteriales	1	1	0	8	26	18	113	82	92	4	14	3	362
7	order	Lactobacillales	10	1	7	6	10	8	13	8	43	14	0	5	125
7	order	Fusobacteriales	4	6	2	10	5	4	13	11	21	8	3	2	89
7	order	Desulfurellales	6	8	10	5	5	5	7	2	2	23	23	24	120
7	order	Fibrobacterales	9	10	8	31	47	47	7	11	3	11	2	5	191
7	order	Deinococcales	0	2	0	7	6	2	2	3	3	14	6	15	60
7	order	Rickettsiales	10	7	7	39	19	31	6	13	13	11	5	1	162
7	order	Bdellovibrionales	39	19	15	44	67	53	6	19	6	30	18	36	352
7	order	Rhodobacterales	5	7	2	9	5	7	4	6	4	2	1	2	54
7	order	Cytophagales	27	32	21	74	60	67	22	48	37	74	41	16	519

7	order	Bacteroidales	16	8	12	17	13	8	25	21	21	94	16	16	267
7	order	Ignavibacteriales	9	0	6	0	1	1	0	0	1	5	21	1	45
7	order	Caldilineales	13	22	7	35	32	30	11	8	9	45	29	37	278
7	order	Ktedonobacterales	0	0	0	0	0	0	62	40	3	0	0	0	105
7	order	Herpetosiphonales	2	7	6	12	12	13	0	0	0	3	2	1	58
7	order	Desulfuromonadales	12	10	3	2	23	6	0	0	0	1	3	4	64
7	order	Actinomycetales	4	1	5	11	4	1	15	8	20	3	7	9	88
7	order	Legionellales	10	17	15	16	11	29	4	8	13	14	15	14	166
7	order	Methylococcales	8	9	8	0	2	1	1	3	3	0	0	0	35
7	order	Pasteurellales	1	0	0	4	1	1	2	4	8	1	3	1	26
7	order	Neisseriales	2	1	3	5	4	0	5	8	5	5	4	2	44
7	order	Rhodocyclales	0	1	2	0	4	1	3	1	4	5	11	1	33
7	order	Thermotogales	0	0	0	9	0	0	0	0	0	0	0	0	9
7	order	Enterobacteriales	0	0	0	1	1	1	2	2	0	9	0	0	16
7	order	Campylobacterales	0	0	0	0	1	2	2	2	2	1	0	2	12
7	order	Desulfobacterales	0	0	0	1	3	0	0	0	1	0	0	0	5
7	order	Thiotrichales	0	1	0	0	1	2	0	0	0	0	0	0	4
7	order	Rubrobacterales	0	0	0	4	0	0	0	0	0	0	0	0	4
7	order	Coriobacteriales	0	0	3	0	0	0	0	0	0	0	0	0	3
7	order	Alteromonadales	2	1	0	0	0	0	0	0	0	0	0	0	3
7	order	Cardiobacteriales	1	0	0	0	0	0	0	0	1	0	0	0	2
7	order	Spirochaetales	1	0	0	0	0	0	0	0	0	0	1	0	2
7	order	Procabacteriales	0	0	0	0	0	0	0	0	0	1	0	1	2
8	family	UnClassified	14714	11855	12489	15869	13284	12799	16298	13133	12408	15270	13038	13723	164880
8	family	Alcaligenaceae	142	107	168	255	82	75	39	58	48	394	506	426	2300

8	family	Phyllobacteriaceae	21	50	1238	2	9	3	2	104	6	12	8	11	1466
8	family	Nitrospiraceae	546	362	350	419	261	283	336	235	236	434	557	505	4524
8	family	Methylophilaceae	380	758	432	47	7	11	49	164	70	3	1	4	1926
8	family	Nocardiaceae	48	220	637	0	2	6	6	32	16	10	6	16	999
8	family	Comamonadaceae	462	786	1341	701	1249	1241	217	863	183	754	365	494	8656
8	family	Bradyrhizobiaceae	40	61	119	47	78	70	360	339	234	21	22	29	1420
8	family	Micrococcaceae	80	123	504	27	48	18	36	80	652	5	6	10	1589
8	family	Sphingomonadaceae	292	285	303	587	531	458	386	353	275	203	145	169	3987
8	family	Pseudomonadaceae	167	230	174	59	18	45	9	41	8	41	7	8	807
8	family	Oxalobacteraceae	143	281	339	178	633	858	106	364	134	132	33	289	3490
8	family	Nocardioidaceae	313	507	540	463	281	267	222	236	107	207	115	156	3414
8	family	Xanthomonadaceae	111	146	101	413	251	245	231	119	223	609	255	347	3051
8	family	Nitrosomonadaceae	532	237	543	427	478	441	346	339	200	271	255	236	4305
8	family	Haliangiaceae	44	33	44	201	109	79	158	110	88	192	192	231	1481
8	family	Burkholderiaceae	5	24	2	57	77	64	36	37	31	2	2	0	337
8	family	Hyphomicrobiaceae	53	59	53	34	31	32	170	116	92	173	197	181	1191
8	family	Intrasporangiaceae	47	86	57	120	209	201	147	120	179	100	96	146	1508
8	family	Gemmatimonadaceae	556	359	405	677	717	706	633	511	535	885	866	873	7723
8	family	Sinobacteraceae	27	63	49	65	21	18	130	107	85	354	211	286	1416
8	family	Chloroflexaceae	21	41	24	51	68	62	69	40	35	196	364	151	1122
8	family	Phaselicystidaceae	7	5	6	12	22	21	138	96	85	52	62	57	563
8	family	Anaerolineaceae	145	196	92	110	77	79	114	79	45	308	377	282	1904
8	family	Moraxellaceae	21	3	162	4	49	73	4	98	5	12	17	42	490
8	family	Micromonosporaceae	2	3	3	6	4	0	107	108	51	89	137	98	608
8	family	Frankiaceae	12	5	1	42	23	10	100	67	40	28	18	19	365

8	family	Acetobacteraceae	36	64	23	77	97	113	86	58	70	159	79	89	951
8	family	Rhodobiaceae	66	66	82	26	26	20	46	33	35	28	66	42	536
8	family	Acidimicrobiaceae	110	113	93	141	54	60	118	75	83	147	99	129	1222
8	family	Chromatiaceae	23	9	24	158	13	21	0	14	2	0	0	0	264
8	family	Xanthobacteraceae	50	38	58	30	43	29	302	249	176	76	103	100	1254
8	family	Solirubrobacteraceae	81	63	63	42	26	32	14	11	5	37	24	39	437
8	family	Pseudonocardiaceae	12	21	8	27	67	63	22	20	14	228	83	138	703
8	family	Cryptosporangiaceae	9	34	5	18	5	7	5	0	0	46	49	58	236
8	family	Flavobacteriaceae	34	27	28	36	26	20	38	31	26	23	27	98	414
8	family	Hyphomonadaceae	0	0	0	0	0	0	7	2	1	38	49	61	158
8	family	Sporichthyaceae	92	86	53	39	21	18	15	9	14	43	32	43	465
8	family	Rhodospirillaceae	39	13	25	2	40	53	90	83	58	7	10	8	428
8	family	Beijerinckiaceae	0	3	0	7	27	18	107	73	95	0	2	0	332
8	family	Planctomycetaceae	370	262	317	286	264	237	296	170	181	411	303	281	3378
8	family	Veillonellaceae	7	2	9	9	0	5	10	13	52	34	6	10	157
8	family	Planococcaceae	57	9	62	0	7	7	0	0	2	0	2	2	148
8	family	Caulobacteraceae	13	17	15	8	32	30	87	47	77	26	20	27	399
8	family	Hydrogenophilaceae	0	2	7	3	4	2	9	2	8	23	33	28	121
8	family	Patulibacteraceae	33	29	20	94	61	58	23	8	13	38	31	34	442
8	family	Phycisphaeraceae	40	37	20	27	25	18	47	36	33	55	82	73	493
8	family	Iamiaceae	44	32	16	45	25	20	45	15	26	98	74	81	521
8	family	Kineosporiaceae	2	8	7	4	25	37	12	18	3	6	1	7	130
8	family	Saprospiraceae	45	28	46	108	71	87	37	27	47	77	45	43	661
8	family	Syntrophaceae	0	0	0	0	3	0	0	0	0	39	9	16	67
8	family	Acidothermaceae	4	3	2	4	1	0	50	33	16	7	3	7	130

8	family	Verrucomicrobiaceae	31	33	18	36	26	24	23	13	35	42	21	24	326
8	family	Polyangiaceae	26	22	23	75	97	61	31	42	49	79	69	60	634
8	family	Acidobacteriaceae	1	1	0	8	26	18	113	82	92	4	14	3	362
8	family	Streptococcaceae	9	1	7	5	9	8	11	8	34	9	0	5	106
8	family	Fusobacteriaceae	3	2	2	3	4	2	7	7	13	7	3	0	53
8	family	Desulfurellaceae	6	8	10	5	5	5	7	2	2	23	23	24	120
8	family	Nakamurellaceae	0	0	0	0	0	0	6	5	11	34	5	15	76
8	family	Opitutaceae	11	16	15	28	26	22	55	44	43	70	41	54	425
8	family	Fibrobacteraceae	9	10	8	31	47	47	7	11	3	11	2	5	191
8	family	Trueperaceae	0	0	0	0	0	0	0	0	0	12	6	15	33
8	family	Bdellovibrionaceae	20	16	8	40	57	47	6	11	6	18	17	28	274
8	family	Rhodobacteraceae	5	7	2	9	5	7	4	6	4	2	1	2	54
8	family	Nannocystaceae	2	5	0	4	0	0	0	2	0	15	25	9	62
8	family	Cytophagaceae	27	31	19	70	56	61	21	48	37	70	38	14	492
8	family	Propionibacteriaceae	1	5	7	10	7	3	3	4	5	18	4	25	92
8	family	Prevotellaceae	12	7	9	9	11	4	18	16	14	23	8	12	143
8	family	Caldilineaceae	13	22	7	35	32	30	11	8	9	45	29	37	278
8	family	Rikenellaceae	0	0	0	0	0	0	0	0	0	28	0	0	28
8	family	Corynebacteriaceae	8	2	6	7	1	3	10	12	14	9	3	1	76
8	family	Herpetosiphonaceae	2	7	6	12	12	13	0	0	0	3	2	1	58
8	family	Microbacteriaceae	16	7	25	2	2	1	2	1	0	2	1	2	61
8	family	Porphyromonadaceae	4	1	3	8	2	4	7	5	7	3	8	4	56
8	family	Actinomycetaceae	4	1	5	11	4	1	15	8	20	3	7	9	88
8	family	Bacteriovoracaceae	19	3	7	4	10	6	0	8	0	12	1	8	78
8	family	Coxiellaceae	7	10	4	13	7	16	4	4	9	11	12	6	103

8	family	Sphingobacteriaceae	7	3	13	7	7	7	10	40	8	9	5	5	121
8	family	Mycobacteriaceae	3	4	1	1	10	5	5	3	1	15	6	8	62
8	family	Bacteroidaceae	0	0	0	0	0	0	0	0	0	40	0	0	40
8	family	Clostridiaceae	11	10	19	0	4	0	9	10	5	35	38	33	174
8	family	Methylococcaceae	7	5	7	0	2	1	1	2	0	0	0	0	25
8	family	Lachnospiraceae	2	1	3	4	4	2	5	4	3	7	1	0	36
8	family	Pasteurellaceae	1	0	0	4	1	1	2	4	8	1	3	1	26
8	family	Neisseriaceae	2	1	3	5	4	0	5	8	5	5	4	2	44
8	family	Methylobacteriaceae	1	0	0	0	0	0	4	18	0	0	2	1	26
8	family	Carnobacteriaceae	1	0	0	1	0	0	2	0	7	0	0	0	11
8	family	Rhodocyclaceae	0	1	2	0	4	1	3	1	4	5	11	1	33
8	family	Streptomycetaceae	2	0	1	2	2	0	9	4	1	0	2	1	24
8	family	Thermotogaceae	0	0	0	9	0	0	0	0	0	0	0	0	9
8	family	Peptococcaceae	3	1	8	0	2	0	0	2	0	3	2	6	27
8	family	Ruminococcaceae	0	0	0	0	1	0	0	0	0	18	1	0	20
8	family	Enterobacteriaceae	0	0	0	1	1	1	2	2	0	9	0	0	16
8	family	Campylobacteraceae	0	0	0	0	1	2	2	2	2	1	0	2	12
8	family	Deinococcaceae	0	2	0	7	6	2	2	3	3	2	0	0	27
8	family	Peptostreptococcaceae	0	0	3	0	2	1	5	2	5	2	2	1	23
8	family	Gallionellaceae	0	0	2	0	1	2	0	3	0	0	0	0	8
8	family	Legionellaceae	3	7	11	3	4	13	0	4	4	3	3	8	63
8	family	Ktedonobacteraceae	0	0	0	0	0	0	0	9	0	0	0	0	9
8	family	Nitrospinaceae	0	0	0	1	3	0	0	0	1	0	0	0	5
8	family	Rickettsiaceae	0	0	0	0	3	6	0	0	0	0	2	0	11
8	family	Cyclobacteriaceae	0	1	0	0	0	1	0	0	0	3	0	0	5

8	family	Erythrobacteraceae	0	2	1	0	0	0	0	0	0	2	0	3	8
8	family	Flammeovirgaceae	0	0	0	0	0	2	0	0	0	1	1	2	6
8	family	Thiotrichaceae	0	1	0	0	1	2	0	0	0	0	0	0	4
8	family	Cystobacteraceae	1	0	0	5	1	1	0	2	0	1	1	1	13
8	family	Coriobacteriaceae	0	0	3	0	0	0	0	0	0	0	0	0	3
8	family	Staphylococcaceae	0	0	0	2	0	0	0	0	0	0	0	1	3
8	family	Alteromonadaceae	2	1	0	0	0	0	0	0	0	0	0	0	3
8	family	Ectothiorhodospiraceae	0	0	0	2	0	0	0	0	0	0	0	0	2
8	family	Bacillaceae	0	0	0	0	0	1	0	2	0	0	0	1	4
8	family	Cardiobacteriaceae	1	0	0	0	0	0	0	0	1	0	0	0	2
8	family	Cellulomonadaceae	0	0	0	0	0	0	1	1	0	0	0	0	2
8	family	Conexibacteraceae	0	0	0	0	0	0	1	1	0	0	0	0	2
8	family	Rhizobiaceae	0	0	2	0	0	0	0	0	0	0	0	0	2
8	family	Geodermatophilaceae	0	0	0	0	0	0	0	0	0	0	0	2	2
8	family	Spirochaetaceae	1	0	0	0	0	0	0	0	0	0	1	0	2
8	family	Aerococcaceae	0	0	0	0	1	0	0	0	0	1	0	0	2
8	family	Brevibacteriaceae	0	0	0	0	0	0	1	0	1	0	0	0	2
8	family	Rhodothermaceae	0	0	0	1	0	0	0	0	0	0	0	1	2
8	family	Paenibacillaceae	0	0	2	0	0	0	0	0	0	0	0	0	2
8	family	Procabacteriaceae	0	0	0	0	0	0	0	0	0	1	0	1	2
8	family	Methylocystaceae	0	0	0	0	0	1	0	1	0	0	0	0	2
9	genus	UnClassified	16294	13003	14373	17386	14734	14432	17496	14253	13131	17090	15026	15791	183009
9	genus	Mesorhizobium	21	49	1238	2	9	3	2	104	6	12	8	10	1464
9	genus	Nitrospira	546	362	350	419	261	283	336	235	236	434	557	505	4524
9	genus	Methylotenera	378	756	423	47	3	7	37	136	62	3	1	4	1857

9	genus	Candidatus Chloracidobacterium	259	303	205	600	534	531	334	242	334	1170	657	794	5963
9	genus	Rhodococcus	47	198	618	0	0	0	0	13	0	1	0	1	878
9	genus	Bradyrhizobium	36	56	112	39	57	47	320	301	206	19	19	27	1239
9	genus	Rothia	4	1	2	7	8	2	7	13	627	2	4	6	683
9	genus	Sphingomonas	235	246	199	414	339	291	343	303	216	131	131	133	2981
9	genus	Pseudomonas	167	229	173	53	11	34	8	41	7	41	6	8	778
9	genus	Aeromicrobium	91	148	303	26	36	24	67	118	37	4	10	12	876
9	genus	Arthrobacter	76	121	499	20	40	16	29	67	25	3	2	4	902
9	genus	Polaromonas	254	416	701	119	453	501	35	483	19	147	23	45	3196
9	genus	Haliangium	44	33	44	201	109	79	158	110	88	192	192	231	1481
9	genus	Massilia	46	90	106	9	41	40	66	173	31	55	7	4	668
9	genus	Limnobacter	5	24	2	57	77	64	36	37	31	2	2	0	337
9	genus	Rhodomicrobium	1	0	2	2	0	0	89	71	34	111	69	90	469
9	genus	Oryzihumus	44	82	56	111	204	198	144	114	174	66	91	137	1421
9	genus	Rhizobacter	51	69	162	83	291	306	18	102	30	6	2	6	1126
9	genus	Albidiferax	26	96	78	23	95	91	5	42	15	10	4	26	511
9	genus	Arenimonas	39	62	50	136	124	122	92	29	62	14	5	8	743
9	genus	Steroidobacter	1	2	3	13	7	8	80	80	63	49	49	53	408
9	genus	Chthoniobacter	148	152	148	195	217	241	232	174	183	385	258	276	2609
9	genus	Roseiflexus	21	41	24	51	67	59	69	40	33	192	359	143	1099
9	genus	Xanthomonas	7	5	3	1	0	0	6	4	4	127	33	65	255
9	genus	Phaselicystis	7	5	6	12	22	21	138	96	85	52	62	57	563
9	genus	Chamaesiphon	1	5	1	113	68	73	0	0	1	0	0	0	262
9	genus	Alkanindiges	10	2	151	1	4	16	4	97	4	6	14	22	331
9	genus	Bryobacter	29	28	17	45	77	54	196	164	179	302	193	189	1473

9	genus	Rhodoplanes	43	38	35	20	24	22	53	28	37	9	18	6	333
9	genus	Candidatus Solibacter	33	25	25	65	113	69	264	286	243	101	135	90	1449
9	genus	Frankia	12	5	1	42	23	10	100	67	40	28	18	19	365
9	genus	Nevskia	0	0	0	0	0	0	2	0	0	68	9	81	160
9	genus	Rhodobium	66	66	82	26	26	20	46	33	35	28	66	42	536
9	genus	Marmoricola	155	207	117	291	92	92	102	84	43	135	80	103	1501
9	genus	Gemmatimonas	164	144	84	311	349	346	346	269	264	290	227	250	3044
9	genus	Nitrosococcus	23	9	24	158	13	21	0	14	2	0	0	0	264
9	genus	Solirubrobacter	81	63	63	42	26	32	14	11	5	37	24	39	437
9	genus	Herbaspirillum	5	11	39	0	84	58	6	98	36	0	0	1	338
9	genus	Pseudonocardia	3	4	1	8	31	31	18	15	10	216	77	126	540
9	genus	Novosphingobium	23	11	59	51	131	103	10	10	9	16	2	7	432
9	genus	Pedomicrobium	6	7	8	6	6	7	22	11	9	14	58	48	202
9	genus	Fodinicola	9	34	5	18	5	7	5	0	0	46	49	58	236
9	genus	Flavobacterium	28	24	20	28	24	17	31	26	16	13	23	81	331
9	genus	Hirschia	0	0	0	0	0	0	3	2	1	35	45	54	140
9	genus	Crossiella	9	17	7	19	35	32	1	1	4	11	5	12	153
9	genus	Hyphomicrobium	0	7	2	0	0	1	1	0	0	33	42	33	119
9	genus	Variovorax	10	32	26	49	68	73	7	23	15	36	10	25	374
9	genus	Acidiphilium	11	20	8	37	77	84	22	9	23	21	1	1	314
9	genus	Nocardioides	64	138	114	135	146	143	51	31	24	49	16	29	940
9	genus	Planctomyces	48	52	31	57	36	33	25	23	21	84	50	34	494
9	genus	Flavisolibacter	15	18	8	49	27	32	0	0	8	79	32	42	310
9	genus	Veillonella	5	2	7	8	0	3	6	7	47	5	5	8	103
9	genus	Sporosarcina	56	9	62	0	7	7	0	0	0	0	0	0	141

9	genus	Panacagrimonas	1	24	13	0	0	0	24	6	9	4	4	2	87
9	genus	Pseudolabrys	2	0	3	0	3	4	63	72	33	15	21	28	244
9	genus	Asanoa	0	0	0	0	0	0	37	19	21	0	0	0	77
9	genus	Pirellula	101	57	89	57	53	45	43	19	27	39	28	27	585
9	genus	Terrimonas	68	25	40	46	1	0	5	3	3	0	19	3	213
9	genus	Rhodanobacter	0	0	0	2	47	43	18	8	21	2	2	0	143
9	genus	Rhodopirellula	9	6	7	12	9	2	1	0	0	11	35	40	132
9	genus	Patulibacter	33	29	20	94	61	58	23	8	13	38	31	34	442
9	genus	Phycisphaera	19	16	5	15	17	12	17	17	10	28	42	31	229
9	genus	Iamia	44	32	16	45	25	20	45	15	26	98	74	81	521
9	genus	Angustibacter	2	3	7	3	13	20	2	8	3	0	1	0	62
9	genus	Schlesneria	8	16	11	19	14	21	7	7	4	49	11	13	180
9	genus	Acidothermus	4	3	2	4	1	0	50	33	16	7	3	7	130
9	genus	Sorangium	17	14	21	65	77	49	25	24	39	69	60	51	511
9	genus	Caulobacter	8	7	6	0	3	2	1	0	7	9	9	7	59
9	genus	Caenimonas	2	7	7	43	18	11	8	41	5	8	0	1	151
9	genus	Nitrosospira	2	4	6	0	9	2	4	7	1	2	0	4	41
9	genus	Streptococcus	9	1	7	5	9	8	11	8	34	9	0	5	106
9	genus	Thermomonas	3	12	5	16	15	18	8	4	9	10	3	2	105
9	genus	Gemmata	39	15	48	19	28	28	42	17	32	27	23	17	335
9	genus	Fusobacterium	3	2	2	3	4	2	7	7	13	7	3	0	53
9	genus	Marinicella	0	0	0	0	0	0	0	1	0	13	17	8	39
9	genus	Duganella	1	0	5	0	2	2	0	1	15	0	1	19	46
9	genus	Humicoccus	0	0	0	0	0	0	1	0	1	34	5	15	56
9	genus	Byssovorax	7	8	0	4	12	7	5	14	3	10	8	7	85

9	genus	Opitutus	11	16	15	28	26	22	55	44	43	70	41	54	425
9	genus	Dokdonella	4	2	0	13	5	11	15	9	20	25	2	4	110
9	genus	Truepera	0	0	0	0	0	0	0	0	0	12	6	15	33
9	genus	Zavarzinella	14	15	7	8	21	9	26	17	21	8	6	10	162
9	genus	Kineosporia	0	3	0	1	12	17	9	8	0	6	0	4	60
9	genus	Bdellovibrio	13	15	7	21	36	30	3	8	2	13	9	13	170
9	genus	Lysobacter	10	9	3	21	2	5	16	6	12	1	0	1	86
9	genus	Nannocystis	2	5	0	4	0	0	0	2	0	15	25	9	62
9	genus	Flexibacter	13	5	13	36	18	19	3	27	12	43	33	6	228
9	genus	Prevotella	12	7	9	9	11	4	18	16	14	21	8	12	141
9	genus	Undibacterium	1	22	4	11	4	2	1	7	4	1	0	7	64
9	genus	Leptolyngbya	0	1	0	11	21	10	0	1	1	2	0	0	47
9	genus	Alistipes	0	0	0	0	0	0	0	0	0	28	0	0	28
9	genus	Corynebacterium	8	2	6	7	1	3	10	12	14	9	3	1	76
9	genus	Filimonas	2	4	0	4	2	6	16	12	14	13	7	14	94
9	genus	Rhodopseudomonas	0	0	0	0	10	12	0	0	0	0	0	0	22
9	genus	Herpetosiphon	2	7	6	12	12	13	0	0	0	3	2	1	58
9	genus	Porphyromonas	3	1	3	8	2	3	7	5	6	1	8	3	50
9	genus	Sediminibacterium	6	4	5	8	2	2	3	6	1	5	3	4	49
9	genus	Phenylobacterium	3	5	4	7	17	18	26	10	7	8	6	14	125
9	genus	Actinomyces	4	1	5	11	4	1	15	8	20	3	7	9	88
9	genus	Luedemannella	2	0	0	0	0	0	13	4	6	1	1	2	29
9	genus	Bacteriovorax	15	1	4	0	6	6	0	8	0	10	0	7	57
9	genus	Ilumatobacter	1	12	2	20	1	2	0	0	0	1	0	0	39
9	genus	Sporocytophaga	10	8	4	11	15	12	1	0	3	7	1	1	73

9	genus	Chroococcidiopsis	0	0	0	5	10	8	0	0	0	0	0	0	23
9	genus	Cellvibrio	0	1	1	6	7	11	1	0	1	0	1	0	29
9	genus	Aquicella	6	7	2	9	4	8	3	4	6	11	12	4	76
9	genus	Pedobacter	7	3	13	7	1	2	2	6	4	0	0	0	45
9	genus	Mycobacterium	3	4	1	1	10	5	5	3	1	15	6	8	62
9	genus	Segetibacter	2	10	1	19	23	23	4	1	20	3	0	0	106
9	genus	Caldilinea	8	15	4	25	20	18	6	4	7	33	20	23	183
9	genus	Mucilaginibacter	0	0	0	0	0	2	5	28	2	0	0	0	37
9	genus	Bacteroides	0	0	0	0	0	0	0	0	0	40	0	0	40
9	genus	Acidocella	2	5	0	3	0	1	1	1	2	2	3	4	24
9	genus	Nordella	2	3	0	0	4	1	3	0	1	1	1	0	16
9	genus	Sandarakinorhabdus	0	1	0	2	2	3	1	4	6	5	1	1	26
9	genus	Perlucidibaca	8	0	6	0	0	0	0	0	0	6	1	4	25
9	genus	Clostridium	11	10	19	0	4	0	9	10	5	35	38	33	174
9	genus	Candidatus Odyssella	0	2	1	11	1	3	3	4	0	2	0	0	27
9	genus	Phascolarctobacterium	0	0	0	0	0	0	0	0	0	14	0	0	14
9	genus	Johnsonella	1	1	1	4	4	2	5	4	3	0	1	0	26
9	genus	Defluviicoccus	0	0	0	0	0	0	0	0	0	3	10	5	18
9	genus	Siphonobacter	2	8	0	6	7	12	2	8	0	10	3	4	62
9	genus	Capnocytophaga	5	2	7	6	2	3	7	5	10	5	0	5	57
9	genus	Haemophilus	1	0	0	1	0	1	1	1	6	0	2	0	13
9	genus	Sporichthya	2	5	2	11	1	1	2	0	0	0	0	0	24
9	genus	Neisseria	0	1	3	2	3	0	5	7	5	5	4	2	37
9	genus	Candidatus Koribacter	0	0	0	0	0	0	4	3	6	0	0	0	13
9	genus	Maribacter	0	0	0	0	0	0	0	0	0	1	3	9	13

9	genus	Cytophaga	0	1	1	10	7	6	2	6	0	2	1	0	36
9	genus	Paucibacter	0	1	3	2	0	0	10	0	2	2	0	9	29
9	genus	Peredibacter	4	2	3	4	4	0	0	0	0	2	1	1	21
9	genus	Microcoleus	0	0	0	0	1	3	0	0	10	0	0	0	14
9	genus	Blastopirellula	3	5	0	0	0	0	0	0	0	5	7	6	26
9	genus	Propionibacterium	0	0	0	1	0	0	1	2	4	2	0	2	12
9	genus	Chitinophaga	0	0	0	0	0	2	0	12	4	2	2	4	26
9	genus	Granulicatella	1	0	0	1	0	0	2	0	7	0	0	0	11
9	genus	Dialister	0	0	0	0	0	0	0	0	0	11	0	0	11
9	genus	Dactylosporangium	0	0	0	4	3	0	1	2	3	2	1	2	18
9	genus	Azospira	0	0	0	0	2	1	3	1	4	0	0	0	11
9	genus	Streptomyces	2	0	1	2	2	0	9	4	1	0	2	1	24
9	genus	Leptotrichia	1	4	0	7	1	2	6	4	8	1	0	2	36
9	genus	Nocardia	0	5	1	0	1	6	2	7	1	0	0	1	24
9	genus	Ramlibacter	0	1	2	2	6	4	0	0	0	0	0	0	15
9	genus	Desulfosporosinus	3	1	8	0	2	0	0	2	0	3	2	6	27
9	genus	Propionicicella	1	0	4	0	0	0	0	0	0	0	0	4	9
9	genus	Devosia	1	3	3	3	1	2	2	2	6	0	0	1	24
9	genus	Subdoligranulum	0	0	0	0	1	0	0	0	0	10	0	0	11
9	genus	Gemella	0	0	0	1	0	0	2	0	6	0	0	0	9
9	genus	Enterobacter	0	0	0	1	0	0	2	2	0	4	0	0	9
9	genus	Hymenobacter	2	8	0	5	6	10	12	5	18	2	0	0	68
9	genus	Singulisphaera	2	5	1	11	16	9	14	12	4	21	6	8	109
9	genus	Campylobacter	0	0	0	0	1	2	2	2	2	1	0	2	12
9	genus	Deinococcus	0	2	0	7	6	2	2	3	3	2	0	0	27

9	genus	Woodsholea	0	0	0	0	0	0	4	0	0	3	4	7	18
9	genus	Peptostreptococcus	0	0	1	0	0	0	3	2	0	2	0	0	8
9	genus	Thiobacillus	0	1	1	0	1	1	4	0	0	0	0	0	8
9	genus	Candidatus Nitrotoga	0	0	2	0	1	2	0	3	0	0	0	0	8
9	genus	Uliginosibacterium	0	0	0	0	0	0	0	0	0	0	7	1	8
9	genus	Aggregatibacter	0	0	0	1	0	0	1	1	2	1	0	1	7
9	genus	Spirosoma	0	1	1	1	2	0	1	2	4	6	0	3	21
9	genus	Nostoc	4	2	2	2	0	0	0	0	5	0	0	0	15
9	genus	Legionella	3	7	11	3	4	13	0	4	4	3	3	8	63
9	genus	Verrucomicrobium	0	2	2	2	2	3	0	0	0	0	0	0	11
9	genus	Acidobacterium	0	0	0	0	0	0	0	2	4	0	0	0	6
9	genus	Humibacillus	0	0	0	0	0	0	0	0	0	6	2	2	10
9	genus	Ktedonobacter	0	0	0	0	0	0	0	9	0	0	0	0	9
9	genus	Candidatus Microthrix	0	1	0	0	2	0	1	0	0	5	6	5	20
9	genus	Selenomonas	2	0	2	0	0	2	3	6	3	2	1	2	23
9	genus	Derxia	0	4	0	2	1	0	0	0	0	0	0	1	8
9	genus	Saxeibacter	0	0	0	0	0	0	1	2	6	0	0	0	9
9	genus	Coxiella	1	3	2	4	3	8	1	0	3	0	0	2	27
9	genus	Candidatus Entotheonella	0	0	0	1	3	0	0	0	1	0	0	0	5
9	genus	Lapillicoccus	0	0	0	1	1	1	0	1	0	4	0	1	9
9	genus	Eikenella	2	0	0	1	1	0	0	1	0	0	0	0	5
9	genus	Rickettsia	0	0	0	0	3	6	0	0	0	0	0	0	9
9	genus	Methylobacter	0	2	4	0	1	1	0	2	0	0	0	0	10
9	genus	Kribbella	0	0	0	0	3	2	1	1	3	4	1	0	15
9	genus	Prosthecobacter	1	1	2	2	4	0	1	0	0	5	2	1	19

9	genus	Rhodovarius	0	0	0	1	1	3	0	0	0	0	0	0	5
9	genus	Friedmanniella	0	2	0	0	4	1	0	0	0	2	2	5	16
9	genus	Rhodovastum	0	1	1	6	0	0	0	0	1	0	0	1	10
9	genus	Aquabacterium	0	0	0	1	0	0	0	1	3	0	1	1	7
9	genus	Amycolatopsis	0	0	0	0	0	0	3	4	0	1	0	0	8
9	genus	Paenisporosarcina	0	0	0	0	0	0	0	0	0	0	2	2	4
9	genus	Leptothrix	0	1	2	2	0	0	2	0	0	5	0	0	12
9	genus	Solitalea	0	0	0	0	0	0	3	0	1	0	0	0	4
9	genus	Janthinobacterium	3	0	1	0	0	0	0	0	0	0	0	0	4
9	genus	Labrys	0	0	0	0	0	0	1	0	0	0	2	1	4
9	genus	Phormidium	0	0	0	2	2	0	0	0	0	0	0	0	4
9	genus	Rugamonas	1	3	0	0	0	0	0	0	0	0	0	0	4
9	genus	Rubrobacter	0	0	0	4	0	0	0	0	0	0	0	0	4
9	genus	Anaeromyxobacter	1	0	0	5	1	1	0	0	0	1	1	1	11
9	genus	Quadrisphaera	0	0	0	0	0	0	0	0	0	0	0	3	3
9	genus	Frigoribacterium	1	1	1	0	0	0	0	0	0	0	1	2	6
9	genus	Subsaxibacter	0	0	0	2	0	0	0	0	0	3	0	0	5
9	genus	Rhodobacter	0	0	0	0	0	0	2	0	1	0	0	0	3
9	genus	Geminicoccus	0	0	0	0	0	0	0	0	0	5	1	1	7
9	genus	Brevundimonas	0	0	0	0	0	0	1	3	1	1	1	1	8
9	genus	Cryobacterium	2	1	3	1	0	0	0	0	0	1	0	0	8
9	genus	Amaricoccus	0	0	0	0	0	0	0	0	0	1	1	1	3
9	genus	Staphylococcus	0	0	0	2	0	0	0	0	0	0	0	1	3
9	genus	Isosphaera	0	1	0	0	2	0	1	1	0	4	0	3	12
9	genus	Phycicoccus	0	0	0	1	1	0	0	0	0	0	1	2	5

9	genus	Methylobacterium	1	0	0	0	0	0	0	2	0	0	2	0	5
9	genus	Azoarcus	0	1	0	0	2	0	0	0	0	0	0	0	3
9	genus	Altererythrobacter	0	2	1	0	0	0	0	0	0	0	0	0	3
9	genus	Actinoplanes	0	0	1	0	0	0	0	0	0	5	0	0	6
9	genus	Candidatus Captivus	0	0	2	1	1	2	0	1	0	0	0	0	7
9	genus	Stenotrophomonas	0	0	0	0	0	1	0	0	0	0	0	2	3
9	genus	Roseomonas	1	0	1	4	0	2	0	0	0	0	0	0	8
9	genus	Faecalibacterium	0	0	0	0	0	0	0	0	0	1	1	0	2
9	genus	Blastochloris	0	0	0	0	0	0	1	0	1	0	0	0	2
9	genus	Megamonas	0	0	0	0	0	0	0	0	0	2	0	0	2
9	genus	Tannerella	1	0	0	0	0	1	0	0	0	0	0	0	2
9	genus	Fibrella	0	0	0	1	1	2	0	0	0	0	0	0	4
9	genus	Crinalium	0	1	0	2	2	1	0	0	0	0	0	0	6
9	genus	Bacillus	0	0	0	0	0	1	0	2	0	0	0	1	4
9	genus	Cardiobacterium	1	0	0	0	0	0	0	0	1	0	0	0	2
9	genus	Ruminococcus	0	0	0	0	0	0	0	0	0	2	0	0	2
9	genus	Paraprevotella	0	0	0	0	0	0	0	0	0	2	0	0	2
9	genus	Actinotalea	0	0	0	0	0	0	1	1	0	0	0	0	2
9	genus	Parabacteroides	0	0	0	0	0	0	0	0	0	2	0	0	2
9	genus	Candidatus Alysiosphaera	0	0	0	0	0	0	0	0	0	1	0	1	2
9	genus	Conexibacter	0	0	0	0	0	0	1	1	0	0	0	0	2
9	genus	Acinetobacter	0	0	1	1	0	0	0	0	0	0	0	0	2
9	genus	Rhizobium	0	0	2	0	0	0	0	0	0	0	0	0	2
9	genus	Treponema	1	0	0	0	0	0	0	0	0	0	1	0	2
9	genus	Abiotrophia	0	0	0	0	1	0	0	0	0	1	0	0	2

9	genus	Brevibacterium	0	0	0	0	0	0	1	0	1	0	0	0	2
9	genus	Rubricoccus	0	0	0	1	0	0	0	0	0	0	0	1	2
9	genus	Paludibacter	0	0	0	0	0	0	0	0	1	0	0	1	2
9	genus	Paenibacillus	0	0	2	0	0	0	0	0	0	0	0	0	2
9	genus	Procabacter	0	0	0	0	0	0	0	0	0	1	0	1	2
9	genus	Aminobacter	0	1	0	0	0	0	0	0	0	0	0	1	2
9	genus	Gillisia	1	0	1	0	0	0	0	0	0	0	0	0	2
9	genus	Methylocystis	0	0	0	0	0	1	0	1	0	0	0	0	2
10	species	UnClassified	15964	14834	15723	17756	15598	15462	17165	15185	13581	18628	15626	16201	191723
10	species	uncultured Acidobacteria bacterium	275	299	286	253	266	272	997	861	847	1222	1120	1596	8294
10	species	uncultured Gemmatimonadetes bacterium	1332	486	1303	1442	1402	1275	548	424	477	463	491	459	10102
10	species	uncultured soil bacterium	689	657	1082	533	366	341	510	414	386	272	272	231	5753
10	species	Rhodococcus erythropolis	44	190	581	0	0	0	0	13	0	1	0	1	830
10	species	uncultured alpha proteobacterium	214	267	218	336	205	209	380	341	253	187	171	143	2924
10	species	uncultured beta proteobacterium	138	104	148	172	274	247	278	279	244	90	79	70	2123
10	species	Arthrobacter oryzae	40	29	336	8	7	1	2	6	0	1	0	3	433
10	species	uncultured Acidimicrobineae bacterium	62	49	45	8	19	21	303	209	124	35	49	45	969
10	species	uncultured actinobacterium	422	228	389	221	138	142	271	222	209	151	123	158	2674
10	species	uncultured Oxalobacteraceae bacterium	57	47	148	12	170	155	107	135	69	26	13	6	945
10	species	uncultured Acidothermaceae bacterium	163	60	128	61	13	16	7	14	2	1	0	0	465
10	species	uncultured Gemmatimonadales bacterium	106	76	127	80	101	88	65	63	81	68	101	89	1045
10	species	uncultured Chloroflexi bacterium	148	84	73	76	79	59	170	82	77	150	114	165	1277
10	species	uncultured bacterium gp4	5	11	6	40	37	43	2	5	4	169	79	90	491
10	species	uncultured gamma proteobacterium	14	16	9	19	8	3	7	5	8	138	33	69	329
10	species	uncultured diatom	16	32	4	322	73	64	13	2	30	26	0	1	583

10	species	beta proteobacterium LH14	24	1	12	9	84	59	0	0	0	3	0	2	194
10	species	uncultured Caldilineaceae bacterium	0	0	1	0	0	0	0	0	0	13	122	59	195
10	species	Antarctic bacterium 3C4	4	1	138	0	1	1	3	37	0	6	12	15	218
10	species	uncultured Burkholderiaceae bacterium	25	11	24	18	33	29	55	31	51	93	92	75	537
10	species	uncultured Verrucomicrobia bacterium	56	78	59	234	237	256	414	355	333	335	254	311	2922
10	species	uncultured Cystobacteraceae bacterium	0	0	0	0	0	0	100	39	29	0	0	0	168
10	species	bacterium Ellin335	6	5	10	3	4	2	28	19	25	27	23	21	173
10	species	uncultured Rubrobacteridae bacterium	25	13	11	5	7	13	43	21	20	0	0	0	158
10	species	uncultured Chlorobi bacterium	3	5	3	5	22	16	1	6	3	106	41	49	260
10	species	uncultured Comamonadaceae bacterium	28	41	45	73	149	86	35	42	31	20	11	18	579
10	species	uncultured Hyphomicrobiaceae bacterium	30	9	20	5	16	5	60	61	33	1	3	5	248
10	species	uncultured Acidobacteriales bacterium	18	4	26	3	93	69	1	1	2	0	0	0	217
10	species	uncultured Bacteroidetes bacterium	50	62	38	114	91	78	76	47	81	116	71	73	897
10	species	uncultured Xiphinematobacteriaceae bacterium	12	26	15	128	23	21	30	28	33	37	10	14	377
10	species	uncultured Nitrosomonadaceae bacterium	35	16	32	10	2	6	18	11	11	4	20	21	186
10	species	uncultured Green Bay ferromanganous micronodule bacterium MNH4	35	11	24	0	40	52	87	79	56	0	0	0	384
10	species	uncultured bacterium 270	6	6	3	6	7	10	74	52	46	17	11	21	259
10	species	uncultivated soil bacterium clone C112	26	19	16	8	12	9	20	9	18	12	25	21	195
10	species	uncultured sludge bacterium S14	0	2	0	1	0	0	2	1	2	50	24	32	114
10	species	uncultured proteobacterium	26	24	29	45	63	65	110	44	97	88	108	107	806
10	species	Sporosarcina globispora	33	8	43	0	0	0	0	0	0	0	0	0	84
10	species	uncultured Chlorophyta	0	0	0	36	35	61	3	1	4	1	2	0	143
10	species	uncultured Sinobacteraceae bacterium	4	25	16	25	4	4	0	0	0	0	0	0	78
10	species	Nocardioides plantarum	8	14	32	5	33	27	0	0	0	0	0	0	119
10	species	beta proteobacterium BP-5	4	1	22	41	1	1	0	0	0	0	0	0	70

10	species	uncultured Xanthomonadaceae bacterium	3	0	0	7	43	40	19	8	21	0	0	0	141
10	species	uncultured Bradyrhizobiaceae bacterium	4	9	3	18	5	8	26	22	9	8	5	10	127
10	species	uncultured Planctomycetales bacterium	8	8	3	6	6	5	6	0	1	6	35	35	119
10	species	uncultured bacterium #0319-7F4	1	0	0	0	3	1	3	3	1	14	33	18	77
10	species	uncultured Frankiaceae bacterium	0	1	0	0	0	0	2	6	1	24	12	25	71
10	species	Actinomycetales bacterium Gsoil 1632	2	6	2	5	5	0	0	0	0	27	10	21	78
10	species	uncultured Acidimicrobidae bacterium	6	12	13	10	1	1	22	9	5	7	6	8	100
10	species	uncultured Planctomycetaceae bacterium	28	8	30	14	2	4	7	4	2	21	11	14	145
10	species	uncultured Myxococcales bacterium	0	2	1	0	8	2	2	1	0	44	13	18	91
10	species	uncultured bacterium 259	6	12	5	23	5	13	6	2	4	20	10	21	127
10	species	Koliella longiseta	0	0	1	9	0	1	10	10	23	3	1	2	60
10	species	uncultured bacterium mle1-48	6	12	5	7	10	8	20	8	8	22	31	34	171
10	species	uncultured Cytophagales bacterium	0	0	0	0	0	0	0	0	0	13	17	13	43
10	species	Streptococcus sanguinis	4	0	4	3	2	4	5	2	11	3	0	2	40
10	species	uncultured Sphingomonadaceae bacterium	3	5	4	29	4	2	1	0	1	2	3	2	56
10	species	Fusobacterium nucleatum	2	2	2	2	2	2	5	5	8	4	3	0	37
10	species	uncultured sludge bacterium H39	14	7	4	4	3	1	4	4	1	34	27	41	144
10	species	planctomycete A-2	12	0	9	1	8	5	7	0	2	1	1	0	46
10	species	Aetherobacter rufus	7	8	0	3	9	4	1	11	2	10	7	7	69
10	species	uncultured Gemmatimonadaceae bacterium	0	0	0	0	18	12	0	1	0	0	0	0	31
10	species	Pseudendoclonium akinetum	1	3	0	20	9	5	0	0	0	0	0	1	39
10	species	uncultured Armatimonadetes bacterium	1	2	0	24	15	9	3	2	0	2	0	1	59
10	species	uncultured bacterium DA008	0	0	0	0	0	0	10	22	11	0	0	0	43
10	species	uncultured Rhodospirillales bacterium	1	7	2	10	0	0	1	0	0	10	7	12	50
10	species	Prevotella intermedia	2	2	1	5	1	1	3	3	2	1	2	2	25

10	species	uncultured prokaryote	6	9	2	24	16	9	1	11	10	17	15	20	140
10	species	uncultured eubacterium WD215	0	0	0	30	6	3	2	4	2	0	0	0	47
10	species	Mesorhizobium loti	2	0	0	0	4	1	0	3	4	6	1	2	23
10	species	uncultured sludge bacterium A24	0	9	3	3	1	0	1	0	0	22	3	3	45
10	species	uncultured candidate division SPAM bacterium	0	0	0	0	5	7	16	1	8	0	0	2	39
10	species	uncultured Acidobacteriaceae bacterium	0	0	0	0	0	0	7	7	8	0	0	0	22
10	species	Prevotella loescheii	2	2	3	3	4	1	9	4	9	2	5	6	50
10	species	Rhodopseudomonas palustris	0	0	0	0	10	12	0	0	0	0	0	0	22
10	species	uncultured Flexibacteraceae bacterium	0	0	0	0	0	0	0	0	0	12	8	2	22
10	species	uncultured Rhodocyclaceae bacterium	6	7	2	8	2	1	4	13	20	2	2	4	71
10	species	uncultured Geobacteraceae bacterium	0	0	0	0	20	4	0	0	0	0	0	0	24
10	species	bacterium Ellin6517	0	0	0	0	0	0	10	10	1	0	0	0	21
10	species	uncultured Sphingobacteria bacterium	0	2	0	1	6	3	4	0	6	0	0	0	22
10	species	Nocardioides islandensis	0	0	0	0	0	0	0	0	0	11	2	13	26
10	species	Bacillaria paxillifer	0	1	0	10	7	2	0	0	0	0	0	0	20
10	species	uncultured Termite group 1 bacterium	6	2	0	7	11	4	3	3	1	3	5	2	47
10	species	uncultured Actinobacteridae bacterium	0	11	0	7	1	0	0	0	0	0	0	0	19
10	species	agricultural soil bacterium SC-I-11	11	5	3	6	5	3	3	5	0	4	1	2	48
10	species	filamentous bacterium Plant1 Iso8	1	2	4	6	1	7	4	3	2	21	9	8	68
10	species	uncultured Legionellales bacterium	0	1	0	0	0	0	1	1	0	8	6	1	18
10	species	Sphingobacteriaceae bacterium BR5-29	0	0	0	0	0	0	5	4	7	4	2	0	22
10	species	uncultured Planctomycetacia bacterium	2	3	1	1	8	13	3	3	1	2	0	3	40
10	species	uncultured Anaerolineales bacterium	0	0	0	2	1	0	1	2	0	8	8	2	24
10	species	uncultured Rubrobacteraceae bacterium	0	0	0	0	0	0	0	0	1	3	4	7	15
10	species	uncultured Polyangiaceae bacterium	3	2	5	2	1	2	0	0	0	0	0	0	15

10	species	Chlorella sorokiniana	0	2	1	5	3	3	0	1	1	1	0	0	17
10	species	Phascolarctobacterium faecium	0	0	0	0	0	0	0	0	0	14	0	0	14
10	species	candidate division TM7 single-cell isolate TM7a	1	1	0	1	1	1	2	2	1	2	1	1	14
10	species	Sporichthya polymorpha	2	4	1	11	1	1	1	0	0	0	0	0	21
10	species	uncultured bacterium #0319-6E22	6	8	4	1	15	6	1	3	3	20	13	16	96
10	species	uncultured endolithic bacterium	3	7	7	10	7	4	2	7	2	6	9	8	72
10	species	Actinomyces odontolyticus	0	0	0	3	2	0	4	3	7	0	3	0	22
10	species	uncultured Nitrospirales bacterium	4	2	2	0	0	0	3	2	0	0	0	0	13
10	species	Pseudomonas syringae	6	4	0	2	0	1	0	0	0	0	0	0	13
10	species	Sorangium cellulosum	0	0	0	4	0	0	0	0	3	4	3	1	15
10	species	uncultured candidate division TM7 bacterium	0	0	0	0	9	5	0	2	0	0	1	1	18
10	species	uncultured cyanobacterium	2	6	5	4	10	13	2	4	12	0	5	0	63
10	species	Capnocytophaga sputigena	2	1	3	0	0	0	1	1	3	1	0	1	13
10	species	uncultured bacterium MK04	2	4	1	4	0	0	0	0	0	1	0	1	13
10	species	uncultured planctomycete	6	10	6	4	3	1	8	3	11	12	16	13	93
10	species	Corynebacterium durum	1	1	1	0	0	0	6	2	5	0	1	0	17
10	species	uncultured Actinomycetales bacterium	0	0	0	0	1	1	5	1	3	0	1	1	13
10	species	Rubrobacteridae bacterium Gsoil 1167	1	1	0	3	1	4	0	0	0	1	0	0	11
10	species	uncultured Pseudomonadales bacterium	2	0	0	1	0	1	1	0	0	9	0	2	16
10	species	uncultured Burkholderiales bacterium	0	0	0	0	2	1	3	1	4	0	0	0	11
10	species	Streptomyces scabrisporus	0	0	0	0	0	0	9	0	1	0	0	0	10
10	species	Armatimonas rosea	0	0	1	2	2	3	0	1	1	0	0	0	10
10	species	uncultured Firmicutes bacterium	6	0	5	6	3	7	9	6	6	0	1	0	49
10	species	Rhodococcus fascians	1	0	9	0	0	0	0	0	0	0	0	0	10
10	species	uncultured bacterium GC55	2	3	4	0	2	0	0	1	3	4	4	7	30

10	species	Clostridium bowmanii	2	0	1	0	3	0	0	0	0	9	3	2	20
10	species	uncultured Caldilineales bacterium	3	4	0	0	2	2	0	2	2	1	2	2	20
10	species	uncultured sludge bacterium A31	3	0	0	2	2	3	0	1	0	3	6	9	29
10	species	Chthoniobacter flavus	1	3	4	1	0	0	0	0	0	0	0	0	9
10	species	uncultured Rhodospirillaceae bacterium	0	0	0	0	0	0	3	5	0	1	0	0	9
10	species	bacterium enrichment culture clone auto142 4W	0	0	0	0	0	0	1	7	1	0	0	0	9
10	species	Actinomyces viscosus	1	0	1	0	0	0	1	2	2	0	1	1	9
10	species	Campylobacter showae	0	0	0	0	1	2	2	1	0	0	0	2	8
10	species	Sphingomonas melonis	0	0	0	1	0	0	0	4	3	0	0	0	8
10	species	uncultured eubacterium WD260	0	0	0	0	0	0	3	3	2	0	0	0	8
10	species	uncultured bacterium GKS2-30	0	0	0	0	0	1	1	0	0	6	0	3	11
10	species	uncultivated soil bacterium clone C105	0	0	2	1	1	0	0	1	0	5	8	5	23
10	species	unidentified eubacterium EA25	1	1	1	7	5	4	9	7	4	0	0	1	40
10	species	uncultured Acidimicrobiales bacterium	0	0	0	1	0	0	0	0	2	2	2	1	8
10	species	uncultured sludge bacterium A12	0	0	0	3	0	1	4	0	0	3	2	1	14
10	species	uncultured sulfur-oxidizing symbiont bacterium	1	2	0	0	0	0	1	2	2	1	1	0	10
10	species	Prevotella saccharolytica	1	1	1	0	0	0	2	2	0	0	0	0	7
10	species	uncultured Frankineae bacterium	0	0	0	0	0	0	3	3	1	0	0	0	7
10	species	uncultured bacterium SBR1071	0	0	0	0	5	2	0	0	0	0	0	0	7
10	species	Planctomycetales bacterium Ellin7224	1	0	0	0	0	0	0	0	0	2	1	3	7
10	species	bacterium Ellin6067	1	2	0	3	2	1	0	3	0	0	5	0	17
10	species	uncultured verrucomicrobium DEV021	0	1	0	0	2	1	0	0	0	2	1	1	8
10	species	Trebouxia aggregata	0	0	0	0	0	0	0	6	0	0	0	0	6
10	species	unidentified rhodophyte PRD01a010B	1	3	6	0	1	0	0	0	0	0	0	0	11
10	species	Leptotrichia trevisanii	0	1	0	1	0	1	0	0	1	1	0	1	6

10	species	Phaeodactylum tricornutum	1	1	0	9	1	1	0	1	9	0	0	0	23
10	species	bacterium TG154	0	0	0	6	0	0	0	0	0	0	0	0	6
10	species	uncultured Desulfuromonadales bacterium	1	2	0	0	0	0	2	0	0	2	3	6	16
10	species	uncultured delta proteobacterium	0	2	5	4	8	3	1	4	1	6	0	0	34
10	species	Clostridium psychrophilum	0	0	0	0	0	0	2	1	1	1	0	0	5
10	species	Eikenella corrodens	2	0	0	1	1	0	0	1	0	0	0	0	5
10	species	uncultured candidate division WS5 bacterium	5	2	4	0	0	0	0	0	0	3	3	1	18
10	species	uncultured Ktedobacteria bacterium	0	0	0	0	0	0	9	0	1	0	0	0	10
10	species	uncultured bacterium 89	0	0	0	0	1	0	2	2	0	0	0	0	5
10	species	bacterium Ellin6537	0	0	0	0	0	0	0	0	0	4	0	1	5
10	species	Chlorella mirabilis	1	3	0	0	1	4	0	0	1	0	0	0	10
10	species	uncultured Verrucomicrobia subdivision 3 bacterium	0	0	1	0	1	0	0	2	1	1	1	0	7
10	species	Nocardioides dilutus	0	0	0	0	0	0	0	0	0	5	0	0	5
10	species	Capnocytophaga gingivalis	0	1	0	1	0	0	0	1	1	1	0	0	5
10	species	Leptolyngbya boryana	0	0	0	1	3	0	0	0	0	0	0	0	4
10	species	uncultured sludge bacterium	2	1	3	3	0	0	3	0	0	1	4	1	18
10	species	Variovorax boronicumulans	0	0	0	1	1	2	0	0	0	0	0	0	4
10	species	uncultured Sphingobacteriales bacterium	0	0	0	0	0	0	2	1	1	0	0	0	4
10	species	Acidobacteria bacterium IGE-015	0	0	1	0	0	0	0	0	0	0	1	2	4
10	species	Chloroidium saccharophilum	1	0	0	2	0	0	0	0	1	0	0	0	4
10	species	uncultured bacterium KF-JG30-B11	2	3	1	1	1	0	3	0	3	0	0	0	14
10	species	Sphingomonas echinoides	1	1	0	0	0	0	1	0	0	1	0	0	4
10	species	uncultured Uromyces	1	2	4	0	0	0	1	0	0	0	0	0	8
10	species	Psychrobacillus psychrodurans	1	0	0	0	0	0	0	0	2	0	0	0	3
10	species	Actinomyces naeslundii	0	0	0	1	0	0	0	0	0	1	0	1	3

10	species	uncultured Clostridiales bacterium	0	0	0	2	0	0	1	0	0	0	0	0	3
10	species	Pythium ultimum	0	0	0	0	0	0	0	0	0	3	0	0	3
10	species	uncultured sludge bacterium H8	0	0	0	0	0	0	0	0	0	1	1	1	3
10	species	Klebsormidium flaccidum	0	0	2	1	0	0	0	0	0	0	0	0	3
10	species	uncultured bacterium C2-12	0	0	0	0	0	0	2	1	0	0	0	0	3
10	species	Staphylococcus pasteuri	0	0	0	2	0	0	0	0	0	0	0	1	3
10	species	Chlorokybus atmophyticus	0	0	0	2	1	0	0	0	0	0	0	0	3
10	species	uncultured Alcaligenaceae bacterium	0	0	0	0	0	0	1	0	0	0	1	1	3
10	species	uncultured eubacterium E1-K14	2	0	0	0	0	0	0	1	0	0	0	0	3
10	species	uncultured candidate division SAM bacterium	0	0	0	0	0	3	3	2	1	0	0	0	9
10	species	uncultured Rubrobacterales bacterium	0	0	0	0	0	0	0	0	0	1	0	2	3
10	species	uncultured sludge bacterium A9	0	0	0	0	0	0	0	0	0	1	0	2	3
10	species	Prevotella micans	0	0	1	0	1	0	0	0	0	0	0	0	2
10	species	uncultured verrucomicrobium DEV045	0	0	0	0	0	0	0	0	0	1	1	0	2
10	species	Bacteroides plebeius	0	0	0	0	0	0	0	0	0	4	0	0	4
10	species	Prevotella marshii	0	0	0	0	1	0	1	0	0	0	0	0	2
10	species	Bacteroides dorei	0	0	0	0	0	0	0	0	0	2	0	0	2
10	species	Iamia majanohamensis	0	0	0	0	0	0	0	0	0	0	0	2	2
10	species	uncultured candidate division SR1 bacterium	0	0	0	0	0	0	0	0	0	0	2	0	2
10	species	uncultured soil bacterium PBS-22	0	0	0	0	0	0	0	1	0	0	1	0	2
10	species	uncultured Methylocystaceae bacterium	0	0	0	0	0	0	1	0	1	0	0	0	2
10	species	uncultured epsilon proteobacterium	0	0	0	0	0	0	0	1	1	0	0	0	2
10	species	uncultured Nocardioidaceae bacterium	0	0	0	0	0	0	0	0	0	1	0	1	2
10	species	uncultured candidate division WS6 bacterium	0	0	0	0	0	0	0	0	0	0	0	2	2
10	species	uncultured bacterium gp6	1	0	1	0	0	0	0	0	0	0	0	0	2

10	species	Actinomyces oris	0	1	1	0	0	0	0	0	0	0	0	0	2
10	species	uncultured bacterium #0319-6A14	0	0	0	0	1	1	0	0	0	0	0	0	2
10	species	uncultured verrucomicrobium DEV114	0	1	0	1	0	0	0	0	0	0	0	0	2
10	species	Campylobacter gracilis	0	0	0	0	0	0	0	1	1	0	0	0	2
10	species	uncultured Nitrospirae bacterium	0	0	0	0	1	1	0	0	0	0	0	0	2
10	species	Cardiobacterium valvarum	1	0	0	0	0	0	0	0	1	0	0	0	2
10	species	Ruminococcus callidus	0	0	0	0	0	0	0	0	0	2	0	0	2
10	species	Deinococcus marmoris	0	0	0	0	0	0	0	0	0	2	0	0	2
10	species	Roseomonas aquatica	0	0	0	1	0	1	0	0	0	0	0	0	2
10	species	Parabacteroides merdae	0	0	0	0	0	0	0	0	0	2	0	0	2
10	species	uncultured bacterium SJA-171	0	0	0	0	0	0	0	0	0	0	0	2	2
10	species	uncultured bacterium 253	0	2	0	0	0	0	0	0	0	0	0	0	2
10	species	Rhizobium giardinii	0	0	2	0	0	0	0	0	0	0	0	0	2
10	species	uncultured Methylococcaceae bacterium	0	0	0	1	1	0	0	0	0	0	0	0	2
10	species	uncultured Verrucomicrobiales bacterium	0	1	0	0	1	0	0	0	0	0	0	0	2
10	species	Rhizobacter fulvus	0	0	0	0	1	1	0	0	0	0	0	0	2
10	species	uncultured marine bacterium	0	0	1	0	0	0	1	0	0	0	0	0	2
10	species	uncultured Bdellovibrionales bacterium	0	0	1	0	0	0	0	0	0	0	0	1	2
10	species	uncultured Sorangiineae bacterium	0	0	0	0	0	0	0	0	0	1	0	1	2
10	species	uncultured Prevotellaceae bacterium	0	0	0	0	0	0	0	1	0	1	0	0	2
10	species	Rubrobacterineae bacterium BR7-21	0	0	0	0	0	0	0	0	0	0	2	0	2
10	species	Bacteroides uniformis	0	0	0	0	0	0	0	0	0	2	0	0	2
10	species	Campylobacter concisus	0	0	0	0	0	0	0	0	1	1	0	0	2
