# Supplementary material for: Diversity and structure of soil bacterial communities in the Fildes Region (maritime Antarctica) as revealed by 454 pyrosequencing
Source: Front Microbiol. 2015 Oct 28;6:1188. doi: 10.3389/fmicb.2015.01188 (PMC4623505; doi:10.3389/fmicb.2015.01188)
Supplement: Supplementary file 4 [file Table4.PDF]

Table S4 | Dissimilarity test (multiple response permutation procedure, MRPP) for significant differences in soil bacterial community composition among the different soil types based on OTU abundance-based Bray-Curtis similarity coefficients.

	Pristine	Seal	Penguin	Human	Pristine vs. Seal vs. Penguin vs. Human
Delta	0.3089	0.9651	0.884	0.9095	-
n[#]	3	3	5	3	-
A-value	-	-	-	-	0.1265
Observed delta	-	-	-	-	0.7836
Expected delta	-	-	-	-	0.8971
Significance	-	-	-	-	0.004

Number of samples in each group.

Based on 999 permutations.
