# Supplementary material for: Diversity and structure of soil bacterial communities in the Fildes Region (maritime Antarctica) as revealed by 454 pyrosequencing
Source: Front Microbiol. 2015 Oct 28;6:1188. doi: 10.3389/fmicb.2015.01188 (PMC4623505; doi:10.3389/fmicb.2015.01188)
Supplement: Supplementary file 5 [file Table5.PDF]

Table S5 |Information on the sequence number and taxonomy of the dominant 50 OTUs in the present study.

OTU name	36-1	36-2	36-3	A1-1	A1-2	A1-3	Q11-1	Q11-2	Q11-3	W2-1	W2-2	W2-3	OTU size	Taxonomy
OTU1	20	32	12	24	19	70	180	97	96	657	364	461	2032	Bacteria(100){superkingdom};Bacteroidetes(100){phylum};Sphingobacteriia(100){class};Sphingobacteriales(100){order};Chitinophagaceae(100);uncultured(No Rank)(100);
OTU2	7	21	5	20	2	6	0	1	4	497	457	913	1933	Bacteria(100){superkingdom};Acidobacteria(100){phylum};Acidobacteria(100){class};RB41(100);uncultured_Acidobacteria_bacterium(89){species};
OTU3	127	36	168	6	705	599	61	33	45	0	0	0	1780	Bacteria(100){superkingdom};Fibrobacteres(100){phylum};Fibrobacteria(100){class};KD2-123(100);uncultured_Gemmatimonadetes_bacterium(99){species};
OTU4	6	1520	4	14	41	61	21	8	24	1	4	1	1705	Bacteria(100){superkingdom};Acidobacteria(100){phylum};Acidobacteria(100){class};RB41(100);
OTU5	393	294	419	123	34	50	51	51	30	0	1	1	1447	Bacteria(100){superkingdom};Actinobacteria(100){phylum};Thermoleophilia(100);Gaiellales(100);Gaiellaceae(100);Gaiella(100);uncultured_soil_bacterium(100){species};
OTU6	100	78	123	180	10	6	32	39	31	194	300	276	1369	Bacteria(100){superkingdom};Proteobacteria(100){phylum};Betaproteobacteria(100){class};Burkholderiales(100){order};Alcaligenaceae(100){family};uncultured(No Rank)(100);
OTU7	18	38	1165	2	4	2	2	96	2	3	5	6	1343	Bacteria(100){superkingdom};Proteobacteria(100){phylum};Alphaproteobacteria(100){class};Rhizobiales(100){order};Phyllobacteriaceae(100){family};Mesorhizobium(100){genus};
OTU8	382	81	323	2	158	149	59	48	29	1	0	0	1232	Bacteria(100){superkingdom};Fibrobacteres(100){phylum};Fibrobacteria(100){class};KD2-123(100);uncultured_Gemmatimonadetes_bacterium(100){species};
OTU9	21	45	17	266	147	181	18	6	24	157	111	214	1207	Bacteria(100){superkingdom};Verrucomicrobia(100){phylum};Spartobacteria(100){class};Chthoniobacterales(100);DA101_soil_group(100);
OTU10	18	38	16	314	184	203	26	25	21	69	63	73	1050	Bacteria(100){superkingdom};Verrucomicrobia(100){phylum};Spartobacteria(100){class};Chthoniobacterales(100);DA101_soil_group(100);uncultured_bacterium(No Rank)(81);
OTU11	29	18	26	43	39	45	40	48	22	150	277	290	1027	Bacteria(100){superkingdom};Nitrospirae(100){phylum};Nitrospira(100){class};Nitrospirales(100){order};Nitrospiraceae(100){family};Nitrospira(100){genus};uncultured_bacterium(No Rank)(94);
OTU12	68	593	276	0	0	0	0	0	0	0	0	0	937	Bacteria(100){superkingdom};Proteobacteria(100){phylum};Betaproteobacteria(100){class};Methylophilales(100){order};Methylophilaceae(100){family};Methylotenera(100){genus};
OTU13	4	4	7	5	13	11	292	242	264	9	26	20	897	Bacteria(100){superkingdom};Proteobacteria(100){phylum};Betaproteobacteria(100){class};SC-I-84(100);
OTU14	19	17	5	6	32	19	1	0	1	318	203	251	872	Bacteria(100){superkingdom};Acidobacteria(100){phylum};Acidobacteria(100){class};Candidatus_Chloracidobacterium(100){genus};uncultured_Acidobacteria_bacterium(84){species};
OTU15	30	48	42	39	34	43	244	184	162	0	0	0	826	Bacteria(100){superkingdom};Acidobacteria(100){phylum};Acidobacteria(100){class};RB41(100);uncultured_Acidobacteria_bacterium(94){species};
OTU16	42	168	536	0	0	0	0	13	0	1	0	1	761	Bacteria(100){superkingdom};Actinobacteria(100){phylum};Actinobacteria(100){class};Corynebacteriales(100);Nocardiaceae(100){family};Rhodococcus(100){genus};Rhodococcus_erythropolis(95){species};
OTU17	0	0	0	0	374	384	0	0	0	0	0	0	758	Bacteria(100){superkingdom};Chloroflexi(100){phylum};JG37-AG-4(100);uncultured_bacterium(No Rank)(100);
OTU18	83	181	91	140	93	104	36	15	3	0	5	0	751	Bacteria(100){superkingdom};Acidobacteria(100){phylum};Acidobacteria(100){class};RB41(100);
OTU19	327	100	165	34	5	3	61	27	18	0	2	1	743	Bacteria(100){superkingdom};Nitrospirae(100){phylum};Nitrospira(100){class};Nitrospirales(100){order};Nitrospiraceae(100){family};Nitrospira(100){genus};uncultured_bacterium(No Rank)(91);
OTU20	10	7	7	30	10	5	17	9	9	208	181	171	664	Bacteria(100){superkingdom};Proteobacteria(100){phylum};Betaproteobacteria(100){class};Burkholderiales(100){order};Comamonadaceae(100){family};
OTU21	25	21	28	16	21	13	270	252	2	0	0	0	648	Bacteria(100){superkingdom};Proteobacteria(100){phylum};Alphaproteobacteria(100){class};Rhizobiales(100){order};Bradyrhizobiaceae(100){family};Bradyrhizobium(98){genus};uncultured_alpha_proteobacterium(90){species};

OTU22	3	1	1	7	5	1	6	12	599	1	4	5	645	Bacteria(100){superkingdom};Actinobacteria(100){phylum};Actinobacteria(100){class};Micrococcales(100);Micrococcaceae(100){family};Rothia(100){genus};Rothia_dentocariosa_ATCC_17931(94);
OTU23	20	49	14	14	86	80	67	31	45	51	90	88	635	Bacteria(100){superkingdom};Proteobacteria(100){phylum};Alphaproteobacteria(100){class};Sphingomonadales(100){order};Sphingomonadaceae(94){family};Sphingomonas(89){genus};
OTU24	137	185	153	42	11	28	4	37	6	27	0	5	635	Bacteria(100){superkingdom};Proteobacteria(100){phylum};Gammaproteobacteria(100){class};Pseudomonadales(100){order};Pseudomonadaceae(100){family};Pseudomonas(100){genus};
OTU25	19	5	17	2	4	2	189	217	155	0	0	0	610	Bacteria(100){superkingdom};Proteobacteria(100){phylum};Betaproteobacteria(100){class};SC-I-84(100);
OTU26	219	133	145	70	14	6	5	4	2	3	2	7	610	Bacteria(100){superkingdom};Actinobacteria(100){phylum};Thermoleophilia(100);Gaiellales(100);Gaiellaceae(100);Gaiella(100);
OTU27	61	19	103	404	0	0	2	4	2	1	0	0	596	Bacteria(100){superkingdom};Fibrobacteres(100){phylum};Fibrobacteria(100){class};KD2-123(100);uncultured_Gemmatimonadetes_bacterium(93){species};
OTU28	193	82	236	39	0	0	12	18	1	0	0	0	581	Bacteria(100){superkingdom};Actinobacteria(100){phylum};Thermoleophilia(100);Gaiellales(100);Gaiellaceae(99);Gaiella(99);uncultured_bacterium(No Rank)(98);
OTU29	162	58	91	60	22	17	73	44	42	2	0	0	571	Bacteria(100){superkingdom};Actinobacteria(100){phylum};MB-A2-108(100);
OTU30	125	112	168	19	1	0	68	36	35	0	0	0	564	Bacteria(100){superkingdom};Chloroflexi(100){phylum};S085(100);uncultured_bacterium(No Rank)(100);
OTU31	1	0	0	10	7	9	2	0	0	221	146	151	547	Bacteria(100){superkingdom};Bacteroidetes(100){phylum};Sphingobacteriia(100){class};Sphingobacteriales(100){order};Chitinophagaceae(100);uncultured(No Rank)(100);
OTU32	0	3	1	1	177	350	0	1	0	0	0	3	536	Bacteria(100){superkingdom};Proteobacteria(100){phylum};Betaproteobacteria(100){class};Burkholderiales(100){order};Oxalobacteraceae(100){family};
OTU33	0	1	0	19	84	75	32	27	19	62	111	79	509	Bacteria(100){superkingdom};Proteobacteria(100){phylum};Alphaproteobacteria(100){class};Rhizobiales(100){order};DUNssu371(100);uncultured_Gemmatimonadetes_bacterium(100){species};
OTU34	147	43	83	5	52	42	67	52	14	0	0	0	505	Bacteria(100){superkingdom};Actinobacteria(100){phylum};Thermoleophilia(100);Gaiellales(100);Gaiellaceae(76);Gaiella(76);
OTU35	50	9	241	5	19	12	50	74	18	1	9	8	496	Bacteria(100){superkingdom};Actinobacteria(100){phylum};Actinobacteria(100){class};Propionibacteriales(100);Nocardioidaceae(100){family};Aeromicrobium(100){genus};
OTU36	8	7	8	55	101	76	82	91	63	0	0	0	491	Bacteria(100){superkingdom};Proteobacteria(100){phylum};Betaproteobacteria(100){class};SC-I-84(100);uncultured_beta_proteobacterium(67){species};
OTU37	0	0	0	0	0	0	0	0	0	156	172	163	491	Bacteria(100){superkingdom};Proteobacteria(100){phylum};Alphaproteobacteria(100){class};Rhizobiales(100){order};DUNssu371(100);uncultured_Gemmatimonadetes_bacterium(100){species};
OTU38	66	76	36	148	62	55	13	6	15	0	0	0	477	Bacteria(100){superkingdom};Proteobacteria(100){phylum};Alphaproteobacteria(100){class};Sphingomonadales(100){order};Sphingomonadaceae(100){family};Sphingomonas(100){genus};uncultured_alpha_ proteobacterium(59){species};
OTU39	132	51	90	6	17	15	58	52	42	0	1	0	464	Bacteria(100){superkingdom};Actinobacteria(100){phylum};Thermoleophilia(100);Gaiellales(100);
OTU40	70	8	68	128	36	34	46	17	37	0	0	0	444	Bacteria(100){superkingdom};Fibrobacteres(100){phylum};Fibrobacteria(100){class};KD2-123(100);uncultured_Gemmatimonadetes_bacterium(100){species};
OTU41	12	34	2	52	62	58	43	40	58	39	16	27	443	Bacteria(100){superkingdom};Acidobacteria(100){phylum};Holophagae(100){class};32-20(100);
OTU42	40	51	11	86	35	51	15	8	10	87	16	30	440	Bacteria(100){superkingdom};Nitrospirae(100){phylum};Nitrospira(100){class};Nitrospirales(100){order};Nitrospiraceae(100){family};Nitrospira(100){genus};
OTU43	4	30	79	22	21	26	10	10	191	13	14	20	440	Bacteria(100){superkingdom};Proteobacteria(100){phylum};Alphaproteobacteria(100){class};Rhizobiales(100){order};Bradyrhizobiaceae(100){family};Bradyrhizobium(100){genus};uncultured_alpha_proteoba cterium(85){species};
OTU44	6	10	8	0	10	12	158	117	115	0	0	0	436	Bacteria(100){superkingdom};Actinobacteria(100){phylum};MB-A2-108(100);uncultured_bacterium(No Rank)(96);

OTU45	182	34	169	11	5	5	9	7	6	0	2	1	431	Bacteria(100){superkingdom};Acidobacteria(100){phylum};Acidobacteria(100){class};RB41(100);
OTU46	39	28	331	7	1	0	0	5	0	0	0	1	412	Bacteria(100){superkingdom};Actinobacteria(100){phylum};Actinobacteria(100){class};Micrococcales(100);Micrococcaceae(100){family};Arthrobacter(100){genus};Arthrobacter_oryzae(96){species};
OTU47	10	14	9	46	61	72	49	60	62	5	7	14	409	Bacteria(100){superkingdom};Verrucomicrobia(100){phylum};Spartobacteria(100){class};Chthoniobacterales(100);DA101_soil_group(100);
OTU48	23	10	18	12	48	34	85	75	67	9	10	17	408	Bacteria(100){superkingdom};
OTU49	0	0	0	0	0	0	0	0	0	158	106	139	403	Bacteria(100){superkingdom};Proteobacteria(100){phylum};Gammaproteobacteria(100){class};Xanthomonadales(100){order};Xanthomonadaceae(100){family};
OTU50	5	12	5	0	4	4	176	111	54	7	8	10	396	Bacteria(100){superkingdom};Actinobacteria(100){phylum};Acidimicrobiia(100);Acidimicrobiales(100){order};TM214(100);uncultured_Acidimicrobineae_bacterium(100){species};
