# Supplementary material for: Diversity and structure of soil bacterial communities in the Fildes Region (maritime Antarctica) as revealed by 454 pyrosequencing
Source: Front Microbiol. 2015 Oct 28;6:1188. doi: 10.3389/fmicb.2015.01188 (PMC4623505; doi:10.3389/fmicb.2015.01188)
Supplement: Supplementary file 6 [file Image1.PDF]

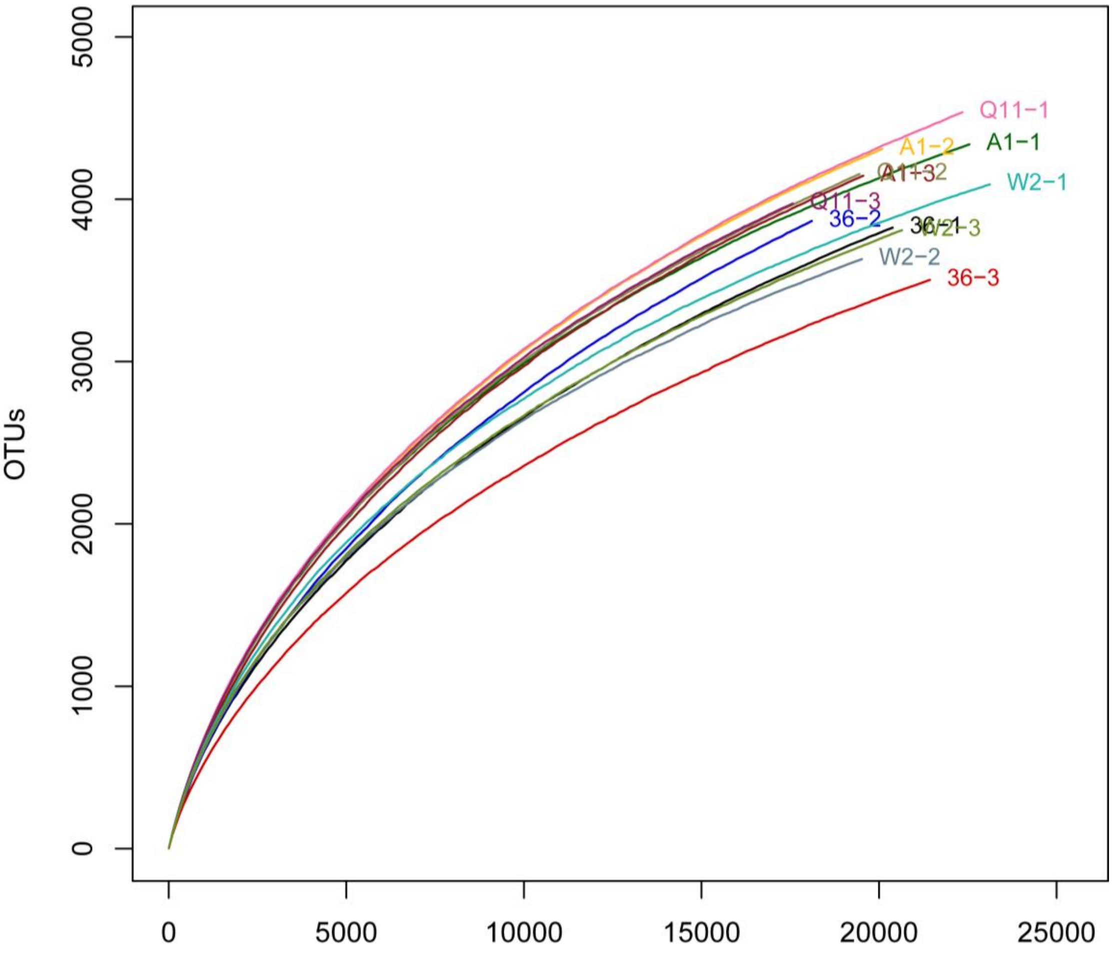

Figure S1 | Rarefaction curves of bacterial 16S rRNA gene sequences from the 12 soil samples in the Fildes Region, calculated by Mothur with a 3% distance cutoff.
