# Supplementary figures and images for: Diversity and structure of soil bacterial communities in the Fildes Region (maritime Antarctica) as revealed by 454 pyrosequencing
Source: Front Microbiol. 2015 Oct 28;6:1188. doi: 10.3389/fmicb.2015.01188 (PMC4623505; doi:10.3389/fmicb.2015.01188)

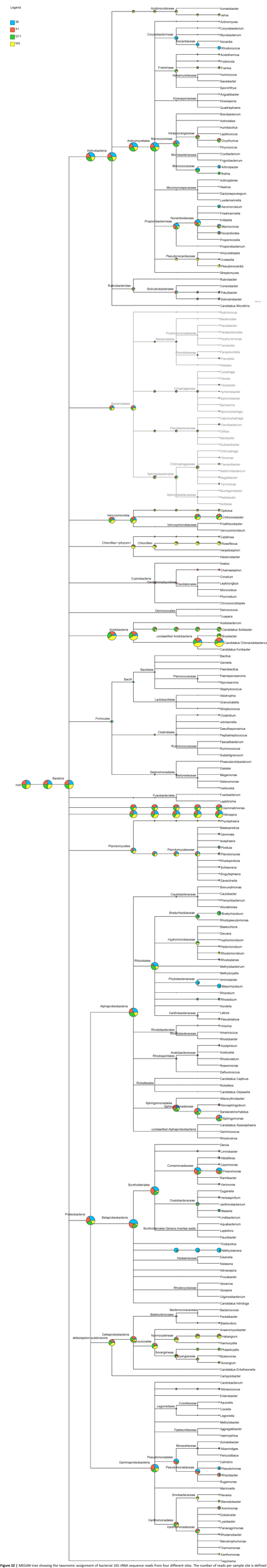

Supplement: Supplementary file 7 [file Image2.PDF]
