# Supplementary material for: Diversity and structure of soil bacterial communities in the Fildes Region (maritime Antarctica) as revealed by 454 pyrosequencing
Source: Front Microbiol. 2015 Oct 28;6:1188. doi: 10.3389/fmicb.2015.01188 (PMC4623505; doi:10.3389/fmicb.2015.01188)
Supplement: Supplementary file 1 [file Table1.PDF]

Table S1 | Relative differences of geochemical properties between the human-, seal- or penguin-colony impacted soils and the pristine soil.

Soil type	Water cont.	pH	Organic C	Organic N	PO_4^{3-}- P	SiO_4^{2-}	NH_4^+-N	NO_2^-- N	NO_3^-- N
human-colony	+37.03	+26.63	+3484.00	+412.00	+5137.68	+47.05	+91.37	+77.82	+429.56
seal-colony	-5.12	+18.90	+144.19	+21.00	+2736.23	+328.95	-9.06	-58.19	-51.54
penguin-colony	+13.70	+12.89	+1467.44	+44.00	+2079.71	+144.95	+37.52	-72.10	+96.02
pristine	0	0	0	0	0	0	0	0	0
